# Super-tough MXene-functionalized graphene sheets

**DOI:** 10.1038/s41467-020-15991-6

**Published:** 2020-04-29

**Authors:** Tianzhu Zhou, Chao Wu, Yanlei Wang, Antoni P. Tomsia, Mingzhu Li, Eduardo Saiz, Shaoli Fang, Ray H. Baughman, Lei Jiang, Qunfeng Cheng

**Affiliations:** 1 Key Laboratory of Bio-inspired Smart Interfacial Science and Technology of Ministry of Education, School of Chemistry, Beihang University, 100191 Beijing, China; 2 School of Transportation Science and Engineering, Beihang University, 100191 Beijing, China; 3 Beijing Advanced Innovation Center for Biomedical Engineering, 100191 Beijing, China; 4 Beijing Key Laboratory of Ionic Liquids Clean Process, CAS Key Laboratory of Green Process and Engineering, State Key Laboratory of Multiphase Complex Systems, Institute of Process Engineering, Chinese Academy of Sciences, 100190 Beijing, China; 5 Key Laboratory of Green Printing, Institute of Chemistry Chinese Academy of Sciences, 100191 Beijing, China; 6 Key Laboratory of Materials Processing and Mold of the Ministry of Education; National Engineering Research Center for Advanced Polymer Processing Technology, Zhengzhou University, 450002 Zhengzhou, China; 7 Center for Advanced Structural Ceramics, Department of Materials, Imperial College London, London, SW7 2AZ UK; 8 Alan G. MacDiarmid NanoTech Institute, University of Texas at Dallas, Richardson, TX 75080 USA; 9 School of Materials Science and Engineering, Zhengzhou University, 450001 Zhengzhou, China

**Keywords:** Mechanical and structural properties and devices, Supercapacitors, Mechanical and structural properties and devices, Two-dimensional materials

## Abstract

Flexible reduced graphene oxide (rGO) sheets are being considered for applications in portable electrical devices and flexible energy storage systems. However, the poor mechanical properties and electrical conductivities of rGO sheets are limiting factors for the development of such devices. Here we use MXene (M) nanosheets to functionalize graphene oxide platelets through Ti-O-C covalent bonding to obtain MrGO sheets. A MrGO sheet was crosslinked by a conjugated molecule (1-aminopyrene-disuccinimidyl suberate, AD). The incorporation of MXene nanosheets and AD molecules reduces the voids within the graphene sheet and improves the alignment of graphene platelets, resulting in much higher compactness and high toughness. In situ Raman spectroscopy and molecular dynamics simulations reveal the synergistic interfacial interaction mechanisms of Ti-O-C covalent bonding, sliding of MXene nanosheets, and π-π bridging. Furthermore, a supercapacitor based on our super-tough MXene-functionalized graphene sheets provides a combination of energy and power densities that are high for flexible supercapacitors.

## Abstract (web summary)

Poor mechanical properties of reduced graphene oxide sheets hinder development of flexible energy storage systems. MXene functionalised graphene oxide with Ti-O-C bonding and additional crosslinking is here reported to dramatically increase toughness for flexible supercapacitors.

## Introduction

Due to ever-increasing demand for mobile devices, particularly portable electrical devices and flexible energy storage systems^1–4^, flexible reduced graphene oxide (rGO) sheets are being considered for such applications. However, the major drawbacks of rGO sheets are their poor mechanical properties and electrical conductivities. The potential for optimizing these sheets for mobile applications has still not been fully realized. One way to reinforce graphene sheets is to introduce different interfacial interactions, such as hydrogen bonding, ionic bonding, π-π bridging, covalent bonding, and a combination of different interfacial interactions^5–7^. A key challenge is to engineer approaches to simultaneously improve the mechanical properties and electrical conductivities of rGO sheets for flexible mobile devices. Recently, new two-dimensional (2D) materials, transition metal carbides (Ti_3_C_2_T_*x*_, MXenes), have been widely investigated due to their high electrical conductivities, large specific surface areas, excellent electrochemical properties, and favorable strengths^8–11^. MXene nanosheets with surface terminated moieties (T_*x*_)^12,13^, such as OH, O, and F, are therefore good candidates for functionalizing GO platelets.

We here demonstrate MXene-functionalized and crosslinked graphene oxide (GO) platelets that were obtained through Ti–O-C covalent bonding. Reaction between the MXene and GO during filtration-based sheet fabrication provided heterosheet bonding. After reduction of the GO, π-π bridging interactions were formed between adjacent rGO platelets using a conjugated molecule (1-aminopyrene (AP)-disuccinimidyl suberate, AD). Hence, synergistic interfacial interactions involving Ti–O-C covalent bonding and π-π bridging occurred in the MXene-functionalized graphene (MrGO-AD) sheet. The MrGO-AD sheet shows ultrahigh toughness (~42.7 MJ m^−3^) and a high failure strain of 12.0%. The tensile strength and electrical conductivity were also improved, reaching as high as ~699.1 MPa and ~1329.0 S cm^−1^. In situ Raman spectroscopy and molecular dynamics simulations reveal that super-high toughness is due to the synergistic interfacial interactions of Ti–O-C covalent bonding and π-π bridging interactions, and the sliding of stacked MXene nanosheets. Furthermore, wide-angle and small-angle X-ray scattering (WAXS and SAXS) show that the orientation of rGO platelets and that the compactness of MrGO-AD sheets is enhanced. Assembled flexible supercapacitors using our MrGO-AD sheet provide a volumetric energy density of ~13.0 mWh cm^−3^ and outstanding flexibility, with ~98% retention of capacitance after 17,000 bending cycles to 180°.

## Results

### Synthesis and characterization of the graphene sheet

**Fig. 1:**
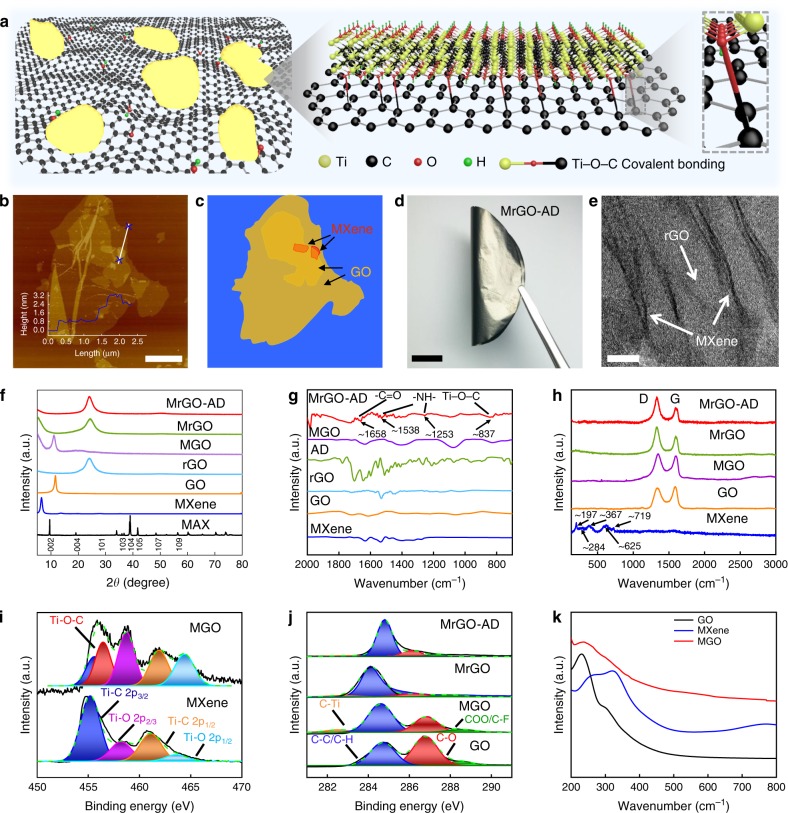
Physical characterization and interactions. **a** Schematic model of MXene-GO platelets showing the formation of Ti–O-C covalent bonding. **b** Atomic force microscope (AFM) of MXene nanosheet-functionalized GO platelets. Scale bar, 5 μm. **c** An illustration of the Mxene-functionalized GO platelets of b. **d**, A photograph of a folded MrGO-AD sheet. Scale bar, 1 cm. **e** A high-resolution transmission electron microscopy (HR-TEM) image of the cross-section of the MrGO-AD sheet. Scale bar, 10 nm. **f** X-ray diffraction (XRD) patterns. **g** Comparisons of Fourier-transform infrared spectroscopy (FTIR) spectra and **h** Raman spectroscopy spectra of obtained sheets. **i** Ti 2p spectra of MXene and MGO sheets. A new peak at a binding energy of 456.5 eV indicates the formation of Ti–O–C covalent bonding between MXene and GO nanosheets. **j** Comparison of C 1s spectra. **k** UV-vis absorption spectra of MXene, GO, and MGO sheets.

The fabrication of a free-standing MrGO-AD sheet is schematically illustrated in Supplementary Fig. 1. The accordion-like MXene (Supplementary Fig. 2) was exfoliated into monolayer MXene nanosheets by using ultrasonic exfoliation. The exfoliated MXene nanosheets have a thickness of ~1.5 nm (Supplementary Fig. 3a) and a lateral size of ~1.5 μm (Supplementary Figs. 4a, b and 5). Our obtained MXene nanosheets show the expected hexagonal structure for the basal planes, high crystallinity, and the absence of nanometer-scale defects that are noticeable in high-resolution transmission electron microscopy (HR-TEM) images and selected-area electron diffraction patterns (Supplementary Fig. 6). As shown in atomic force microscope (AFM) and scanning electron microscope (SEM) images, the obtained GO platelets have a thickness of ~1.0 nm and a lateral size of ~16.0 μm (Supplementary Figs. 3b and 4c, d). The MXene nanosheets, with abundant oxygen-containing functional groups, react at room temperature with the GO platelets to form covalently crosslinked MXene-GO platelets (Fig. 1a–c), which is evidenced by zeta potential changes (Supplementary Fig. 7). As shown in Fig. 1a, the Ti–O-C covalent bonding was formed at the MXene/GO heterointerface via nucleophilic substitution and dehydration reaction (Supplementary Fig. 8). Functional groups of –C=OH^+^ and –C–OH_2_^+^ are first formed in solution with H^+^ ions, due to the abundant oxygen-containing functional group (–COOH and –OH) of GO platelets. As the reaction continues, the MXene-Ti–O^−^ in solution attacks the C of –C=OH^+^ and –C–OH_2_^+^, resulting in the removal of H_2_O from the GO platelets. Finally, the Ti–O–C covalent bonding is formed^14^. The covalent bonding in Ti–O–C is critical for assembling the hybrid architecture, thereby reinforcing the linking of the MXene and GO nanosheets and increasing charge transport.

After filtration, the MGO sheets were further chemically reduced by hydroiodic acid (HI) and immersed in a *N*,*N*-dimethyl formamide (DMF) solution containing long-chain AD molecules, thereby providing π-π bridging interactions between neighboring rGO platelets. Flexible MrGO-AD sheet were thus obtained (Fig. 1d). Elemental mapping spectra by energy-dispersive spectroscopy (for C, O, Ti, and N) in the cross-section of a MrGO-AD sheet show uniform distribution of long-chain AD molecules and MXene nanosheets (Supplementary Fig. 9). These results further confirm that the MXene nanosheets have been successfully introduced within the layers of rGO platelets. HR-TEM images further reveal this insertion of few-layer stacked MXene layers within the volume of rGO platelets (Fig. 1e and Supplementary Fig. 10).

The content of MXene nanosheets in the MGO sheets was determined by thermogravimetric analysis (TGA) (Supplementary Fig. 11 and Supplementary Table 1) (See Supplementary Note 1). All samples were assembled by using basically the same method, and are termed MGO-I, MGO-II, MGO-III, MGO-IV, and MGO-V, depending upon the weight percent of MXene (5.5, 9.9, 17.7, 29.9, and 45.1 wt%, respectively). Although the Supplementary Table 1 provides data for samples containing various concentrations of MXene, this text will refer principally to the MGO-III sheet (containing 17.7 wt% MXene) that provided optimized properties. In the text these sheets will be referred to as MrGO and MGO sheets. Reference samples, such as GO, rGO, rGO-AD, and MGO-AD sheets (where the weight ratio of MXene nanosheets and AD molecules is consistent with MGO-III and rGO-AD), were also fabricated by the same method.

XRD patterns show that MXene sheets have a characteristic 2*θ* peak for CuKα radiation at ~6.30°, which corresponds to a *d-spacing* of 14.64 Å (Fig. 1f and Supplementary Fig. 12). The XRD results indicate that MXene nanosheets (synthesized from Ti_3_AlC_2_) have been successfully exfoliated^15^. The *d-spacing* of the MGO sheet increases to 7.97 from 7.53 Å for neat GO sheets, which suggests that interlamellar insertion of MXene nanosheets into GO sheets has occurred. After being reduced by HI, the *d-spacing* of MrGO sheet decreased to 3.64 Å. This decrease can be attributed to removal of oxygen-containing functional groups from GO platelets. Moreover, compared with the *d-spacing* of MrGO sheet, the *d-spacing* of MrGO-AD sheet increases to 3.68 Å (Supplementary Table 2) due to the introduction of long-chain AD molecules.

Note that in Fig. 1f for MGO and MrGO containing 17.7 wt% MXene that there is the beginning of broad diffraction scattering at low diffraction angles (below the sharp diffraction peak due to the periodicity of neat MXene). Since this scattering increases with increasing MXene content and since the scattering contribution from a MXene layer is much greater than from a rGO layer, we assume that this scattering is due to various localized regions in which one or more MXene layers are associated with rGO layers.

**Fig. 2:**
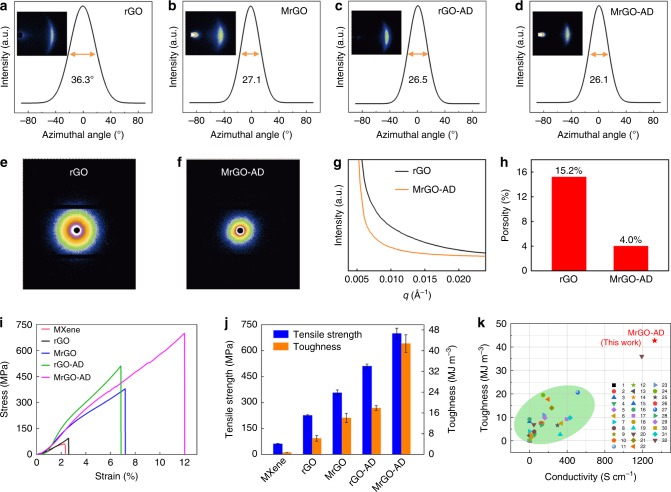
Mechanical characterization. **a**–**d** Azimuthal scan wide-angle X-ray scattering (WAXS) patterns (for a rotating anode X-ray source) showing the full width at half maximum (FWHM) of **a** rGO. **b** MrGO. **c** rGO-AD. d, MrGO-AD. **e**, **f** The corresponding small-angle X-ray scattering (SAXS) patterns for rGO and MrGO-AD. **g** Comparison of SAXS scattering intensities for rGO and MrGO-AD. These results indicate a decrease in porosity in going from rGO to MrGO-AD. **h** Bar graphs comparing the porosity of rGO and MrGO-AD. **i** Typical tensile stress–strain curves for the investigated sheets. **j** Bar chart comparing the toughnesses and conductivity of the obtained sheets. **k** Comparison of tensile strength and toughness MrGO-AD sheet with that of other graphene-based sheets.

New peaks at ~837 cm^−1^ for the MGO, MrGO, and MrGO-AD sheet further confirm the formation of Ti–O–C bonding^16^, which can be seen in the Fourier-transform infrared spectra of Fig. 2g and Supplementary Fig. 13. Furthermore, compared with the spectra of MrGO sheet, some new characteristic peaks appear at wavenumbers of ~1253, ~1538, and ~1658 cm^−1^ in the MrGO-AD sheet, which are attributed to –NH– and –C=O of AD molecules^17^.

Raman spectroscopy (Fig. 1h) shows that the as-prepared MXene sheets have the typical A_1g_ symmetry out-of-plane vibrations of Ti and C atoms (at 197 and 719 cm^−1^) and the E_g_ group vibrations of in-plane modes of Ti, C, and surface functional group atoms (at 284, 367, and 625 cm^−1^). These vibrations are expected for the exfoliated MXene. The ratio of *I*_*D*_ to *I*_*G*_ of the MGO sheet increases to ~1.2, from that of ~0.9 for neat GO sheet (Supplementary Table 3), which is attributed to the reaction with MXene nanosheets. Also, this ratio sharply increases to ~1.6 for MrGO and MrGO-AD sheet, due to reduction by HI. The G-band peak of MGO (at ~1600 cm^−1^) shifts 13 cm^−1^ from the G-band peak (~1587 cm^−1^) of neat GO sheet (Supplementary Fig. 14), indicating the formation of Ti–O–C bonding between MXene and GO nanosheets^18^.

X-ray photoelectron spectroscopy (XPS) (Supplementary Fig. 15) shows the appearance of Ti and N elements, indicating that MXene nanosheets have reacted with rGO platelets. The Ti 2p spectra of pristine MXene sheets (Fig. 1i) display several peaks at binding energies of 455.2, 458.2, 461.1, and 463.6 eV, which are assigned to the bonds of Ti–C 2p_3/2_, Ti–O 2p_3/2_, Ti–C 2p_1/2_, and Ti–O 2p_1/2_, respectively. In contrast, the Ti–O 2p spectra of the MGO sheet show a slight shift to higher binding, from 458.2 to 458.8 eV and from 463.6 to 464.4 eV. Simultaneously (Fig. 1i and Supplementary Fig. 16), a new peak at the binding energy of 456.5 eV was observed. All these results indicate the formation of Ti–O–C covalent bonding between MXene and GO nanosheets, which is consistent with the results of FTIR and Raman spectra^19^. In addition, the atomic percentage of C–O decreases to 30.2% for the MGO sheet (from 51.7% for neat GO sheets), according to C 1s spectra presented in Fig. 1j and Supplementary Table 4. This further indicates the reaction between GO platelets and MXene nanosheets. The UV-vis spectra (Fig. 1k) of GO provides characteristic peaks at 230 and 300 nm, which are attributed to the π-π* transition of C=C and the n-π* transition of carboxyl functional groups. The characteristic peak at 230 nm for GO sheet has red-shifted to 238 nm for MGO, and the shoulder peak at about 300 nm has weakened (Fig. 1k).

### WAXS and SAXS analysis of graphene sheets

Several recent methods, such as the fluidics-enabled assembly or the introduction of interfacial interactions within graphene sheets, have been used for aligning graphene platelets and thereby increasing the tensile strength and toughness of graphene sheet^20^ and fiber^21^. Based on WAXS results (Fig. 2a–d), our MXene-functionalized graphene sheet shows improvement in the alignment degree of graphene platelets. The full width at half maximum (FWHM) of the azimuthal scan for a MrGO sheet decreased to 27.1°, compared with 36.3° for neat rGO sheet. In addition, when the rGO platelets were chemically crosslinked by AD molecules via π-π bridging interactions, the FWHM of rGO-AD sheet was further decreased to 26.5°. The MrGO-AD sheet shows the lowest FWHM (26.1°). Hence, the obtained MrGO, rGO-AD, and MrGO-AD sheets have higher orientation degrees (84.9%, 85.3%, and 85.5%, respectively) than the neat rGO sheet (79.8%), as shown in Supplementary Table 5 (See Supplementary Note 2). Furthermore, the porosity (Supplementary Fig. 17) of the resultant MrGO-AD sheet (calculated by comparing the observed densities with those observed for graphene, MXene, and AD) is reduced to 4.0%, compared with the porosity of the rGO sheet (15.2%), the rGO-AD sheet (8.0%), and the MrGO sheet (5.2%) (See Supplementary Note 3). This low porosity of the MrGO-AD sheet compared to the rGO is reflected in the much lower low-angle x-ray diffraction scattering intensity of the MrGO-AD sheet (Fig. 2g)^22,23^.

### Mechanical properties of the graphene sheets

Stress-strain curves for these sheets are presented in Fig. 2i. Neat GO sheet has a low tensile strength (82.2 ± 1.8 MPa), failure strain (2.6 ± 0.3%), and toughness (0.9 ± 0.05 MJ m^−3^), due to the weak interfacial interactions between GO platelets. When MXene nanosheets are covalently bonded to GO nanosheets, the resulting MGO sheet has a tensile strength of 226.3 ± 4.3 MPa (4.4 ± 0.7% strain) and a toughness of 6.2 ± 1.1 MJ m^−3^, which are 2.8 times and 6.9 times higher than for neat GO sheet. After further reduction by HI, the tensile strength of the MrGO sheet was 379.2 ± 2.5 MPa, the failure strain was 7.2 ± 0.3%, and the toughness was 14.2 ± 1.7 MJ m^−3^. After being chemically crosslinked with AD molecules via π-π bridging interactions, MrGO-AD sheet has a tensile strength of 699.1 ± 30.6 MPa, a failure strain of 12.0 ± 0.7%, and a ultrahigh toughness of 42.7 ± 3.4 MJ m^−3^ (Fig. 2j), which are 4.2 and 17.8 times higher than neat rGO sheet (a tensile strength of 165.8 ± 1.1 MPa and a toughness of 2.4 ± 0.4 MJ m^−3^). Hence, the introduction of AD molecules to crosslink the adjacent graphene platelets results in remarkable improvement of mechanical properties of the graphene sheet.

The enhanced mechanical properties of MrGO-AD sheet are attributed to the synergetic effects of interactions between MXene nanosheets and rGO platelets and between the long-chain AD molecules and the rGO platelets. These interactions provide a high orientation degree and a low porosity for the sheets. The weight content of MXene nanosheets strongly affects the toughness and tensile strength of the resulting MrGO-AD sheet. The MrGO and MGO sheets show the highest tensile strength and toughness for the addition of 17.7 wt% of MXene nanosheets (Supplementary Figs. 18, 19d, and 20d, and Supplementary Table 6). Increasing the content of MXene to beyond 17.7 wt% decreases strength, failure strain, and toughness. More detailed curves and complete data for tensile strength and toughness are presented in Supplementary Figs. 18–20 and Supplementary Tables 6 and 7.

For comparison of mechanical properties, we also prepared graphene-MXene sheet by adding multilayer MXene nanosheets. When the multilayer MXene crystals were added, the solution dispersity was worse than when single-layer MXene nanosheets were added. According to the AFM image in Supplementary Fig. 21, the thickness of multilayer MXene is ~4.5 nm, which is about three layers of MXene nanosheets. The graphene-MXene sheets including multilayer MXene nanosheets (named as MrGO-AD-m) were prepared using the same fabrication process. The obtained samples were then crosslinked with AD molecules into MrGO-AD-m sheet. The stress–strain curves and fracture morphology of MrGO-AD-m sheets are shown in Supplementary Figs. 22 and 23. The tensile strength and toughness of MrGO-AD-m, listed in Supplementary Tables 6 and 7, are ~292.3 MPa and 7.3 MJ m^-3^, respectively. These values are lower than MrGO-AD sheet with the tensile strength of ~699.1 MPa and toughness of ~42.7 MJ m^−3^. The stability in water of neat rGO, MrGO, and MrGO-AD sheets was evaluated, as shown in the newly added Supplementary Fig. 24. This stability was evaluated under very harsh conditions (ultrasonic processing using 100 W, 4.5 KHz ultrasound for 0–7 h). The rGO sheet disintegrated after ultrasonic treatment for 3 h, while the MrGO-AD sheet remained intact during ultrasonic processing for 7 h (as shown in Supplementary Fig. 24).

Our MXene-functionalized graphene sheets also provide high electrical conductivities (Supplementary Table 8), due to the high electrical conductivity of MXene^8^. Neat rGO sheets has an electrical conductivity of 335.8 S cm^−1^. After functionalization by MXene nanosheets, the conductivity of the MrGO sheets increased with increasing content of MXene nanosheets. After being chemically crosslinked with AD molecules, the electrical conductivity of the MrGO sheet increased from 1036.3 ± 5.4 S cm^−1^ to 1329.0 ± 15.9 S cm^−1^ for the MrGO-AD sheet (due to the resulting lower porosity and higher orientation of the MrGO-AD sheet. Our MXene-functionalized graphene sheets provide ultrahigh toughnesses, high tensile strengths, and high electrical conductivities, which provide advantages over other reported graphene sheets^17,20,24–49^, as shown in Fig. 2k and Supplementary Table 9.

### In situ Raman spectroscopy analysis of the graphene sheets

**Fig. 3:**
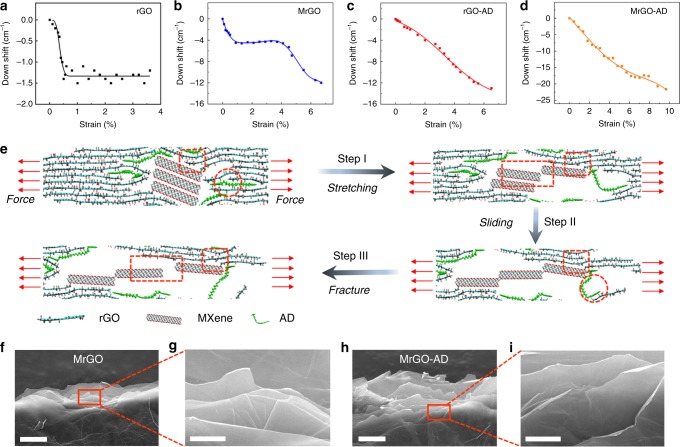
Toughening mechanisms of graphene sheets. **a**–**d** The strain dependence of the downshift in Raman G-band frequency rGO, MrGO, rGO-AD, and MrGO-AD sheets. **e** The proposed fracture mechanism of the MrGO-AD sheet, according to molecular dynamics simulations. Ti–O-C covalent bonding between MXene nanosheets and rGO platelets (elliptical region), π-π bridging interactions between AD molecules and rGO platelets (circular region), and sliding between MXene nanosheets (rectangle region). **f**–**i** Fracture morphology of the side views of MrGO (**f**, **g**) and MrGO-AD (**h**, **i**) sheets. Scale bar, 5 μm (**f**, **h**) and 1 μm (**g**, **i**).

The degree of stress transfer to rGO platelets during mechanical strain (before the end of this transfer because of plastic deformation) is an important metric. We here use the strain-induced downshift in Raman G-band frequency to understand the stress that is supported by the graphene and how it depends on the applied strain. The Raman results for rGO (Fig. 3a) show that there is a small downshift in Raman frequency (1.2–1.4 cm^-1^) at up to 0.5% strain) before a plateau region appears, where there is no further increase in stress transfer to the graphene with further increase in tensile strain. The Raman results for MrGO (Fig. 3b) are more complicated. There is a Raman downshift of 5.3 cm^−1^ before the onset of a plateau region at 0.9% strain, a plateau at between this strain and 4.3% strain, and then further downshift of Raman frequency by 6.7 cm^−1^ before the sample broke at 6.7% strain (Fig. 3b). The type of behavior that is most desirable is shown for both rGO-AD and the MrGO-AD (Fig. 3c,d), where there is continuously increasing stress transfer to the rGO (providing Raman downshifts before rupture of 13.0 and 21.6 cm^−1^, respectively). One interpretation of these results is that the plateau region in the stress–strain curve for MrGO arises because of sliding within the few nanosheet stacks of MXene that are inserted in the volume of the rGO platelets, and that this sliding is arrested for the MrGO-AD because of the π-π bridging provided by the AD. These results can be compared with the mechanical properties of these rGO, MrGO, rGO-AD, and MrGO-AD sheets (with tensile strengths of 165.8, 379.2, 510.4, and 699.1 MPa, respectively, and toughnesses of 2.4, 14.2, 17.9, and 42.7 MJ m^−3^, respectively). These strengths and toughnesses increase in the same order as does the total Raman downshift before sheets failure (1.4, 12.0, 13.0, and 21.6 cm^−1^, respectively for the above list).

### Molecular dynamics simulations of the stretching process

**Fig. 4:**
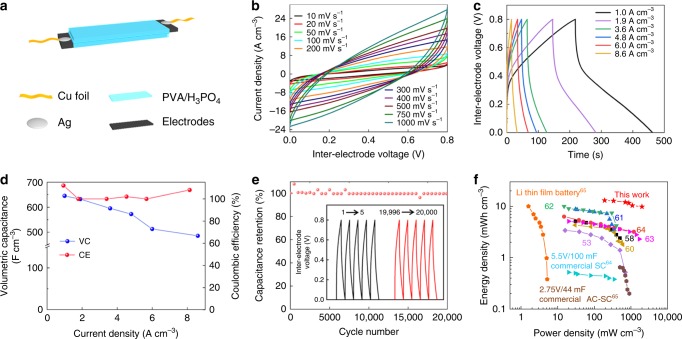
Electrochemical characterization of the supercapacitor. **a** Illustration of the components of a flexible supercapacitor based on MrGO-AD sheets. **b** Cyclic voltammetry (CV) curves at scan rates of 10 to 1000 mV s^−1^ for the MrGO-AD supercapacitor. **c** GCD curves for the MrGO-AD sheet supercapacitor. **d** Volumetric capacitance of the MrGO-AD sheet supercapacitor for current densities from 1.0 to 8.6 A  cm^−3^. **e** Capacitance retention during the cycling of a MrGO-AD supercapacitor. **f** Volumetric energy density and power density of the MrGO-AD supercapacitor compared with alternative energy storage systems.

Molecular dynamics simulations are applied to reveal the synergetic toughening mechanism of MXene-functionalized graphene sheet. As shown in Fig. 3e and Supplementary Figs. 25 and 26, compared with the rGO-AD and MrGO sheets, the MrGO-AD sheet presents a unique synergistic toughening process, such as a plastic deformation process with sliding between MXene nanosheets (rectangle region), and arrest crack propagation process of π-π bridging interactions between AD molecules and rGO platelets (circular region) and bridging of Ti–O-C covalent bonding between MXene nanosheets and rGO platelets (elliptical region). When the stretching procedure starts, the wrinkled rGO platelets are first straightened and the microcrack of adjacent rGO platelets is initiated attributed to the breakage of weak interactions. According to the first-principle calculations in Supplementary Fig. 27, the MXene nanosheets first slide past each other, and then Ti–O–C covalent bonding between MXene and rGO platelets is broken. Therefore, with increasing loading to stretch, MXene nanosheets slide past each other due to the strong Ti–O–C covalent bonding crosslinked with rGO platelets (Step I). The plastic deformation arises mainly from the sliding between MXene nanosheets, which is consistent with the obtained platform of in situ Raman spectroscopy (Fig. 3b). Meanwhile, the long-chain AD molecules crosslinking with rGO platelets via π-π bridging interactions are stretched and further arrest crack propagation before completely fracturing. As a result, the MXene nanosheets are separated from each other and the AD molecules are broken (Step II, III). With further stretching, the Ti–O–C covalent bonding between MXene nanosheets and rGO platelets is broken to arrest crack propagation for promoting toughness until complete fracture of the sheets (Step III) (Supplementary Fig. 26) (See Supplementary Note 4). The fracture morphology of MrGO sheet shows a smooth curvature of rGO platelets (Fig. 4f, g and Supplementary Figs. 28j and 29j), while the MrGO-AD sheet presents obviously curled edges after the pull-out of rGO nanosheets (Fig. 4h, i and Supplementary Figs. 28p and 29p) due to strong synergistic interactions. The corresponding fracture morphologies of other sheets are shown in Supplementary Figs. 28 and 29.

### MXene-functionalized graphene sheet for supercapacitors

Besides high toughness, tensile strength, and electrical conductivity, our MrGO-AD sheet provided potentially useful electrochemical performance. For evaluating this aspect, both MrGO-AD sheets and MrGO sheets were evaluated as symmetric-electrode, flexible supercapacitors using poly(vinyl alcohol) PVA/H_3_PO_4_ as a gel electrolyte, as shown in Fig. 4a. The electrodes used were 5-μm-thick, 2-cm-long, and 1-cm-wide MrGO-AD or MrGO. Cyclic voltammetry (CV) scans for an assembled supercapacitor based on MrGO-AD are shown in Fig. 4b. For potential scan rates between 10 and 750 mV s^−1^, the coulombic efficiency was close to 100%. From galvanostatic charge-discharge (GCD) curves (Fig. 4c), the MrGO-AD sheets in this supercapacitor provided a volumetric capacitance of 645 F cm^−3^ at the current density of 1.0 A cm^−3^, and retained 75.3% of this capacitance for a current density of 8.6 A cm^−3^ with the coulombic efficiency of ~100% (Fig. 4d). The MrGO sheet capacitor provided lower performance. Specifically, the MrGO sheet had a volumetric capacitance of 543 F cm^-3^ for a current density of 1.0 A cm^−3^, and a capacitance retention of 36.2% for a current density of 8.6 A cm^−3^ (Supplementary Fig. 30b, c).

The MrGO-AD sheet supercapacitor provided ~100% capacitance retention after 20,000 cycles, as shown in Fig. 4e. The Ashby plot^50–63^ of mechanical strength versus gravimetric capacitance in Supplementary Fig. 31 and Supplementary Table 10 shows that the MrGO-AD sheet provides remarkable integration of tensile strength and capacitance, compared with other reported supercapacitor electrodes. These alternative include rGO/MnO_2_ film^50^, rGO/polypyrrole film^51^, rGO/cellulose film^52^, rGO wire/CNT fiber^53^, rGO/PANI film^54^, rGO aerogel foam^55^, rGO film^56^, sulfonated aramid-graphene film^57^, graphene/cellulose fiber^58^, rGO/Mn_3_O_4_ film^59^, rGO/aramid film^60^, rGO/PANI fiber^61^, rGO/CNT film^62^, and rGO/MXene fiber^63^. The results indicate that an optimized trade-off between mechanical properties and capacitance was obtained. This combination of high strength (and toughness) with high electrochemical performance might be useful for supercapacitors that provide a structural function, such as supercapacitors in micro-air vehicles or gliders.

Since the solid gel electrolyte in the present device served as temporary packaging, the volume of the entire device was principally that of the MrGO-AD electrodes and the gel electrolyte. In the Fig. 4f Ragone plot for flexible supercapacitors, where we compare the volumetric energy density and power density of the present complete supercapacitor with these metrics for other supercapacitors, all devices (except for four described commercial products) do not have packaging. The references and presented data of Fig. 4f are provided in Supplementary Table 11^53,58,60–65^.

**Fig. 5:**
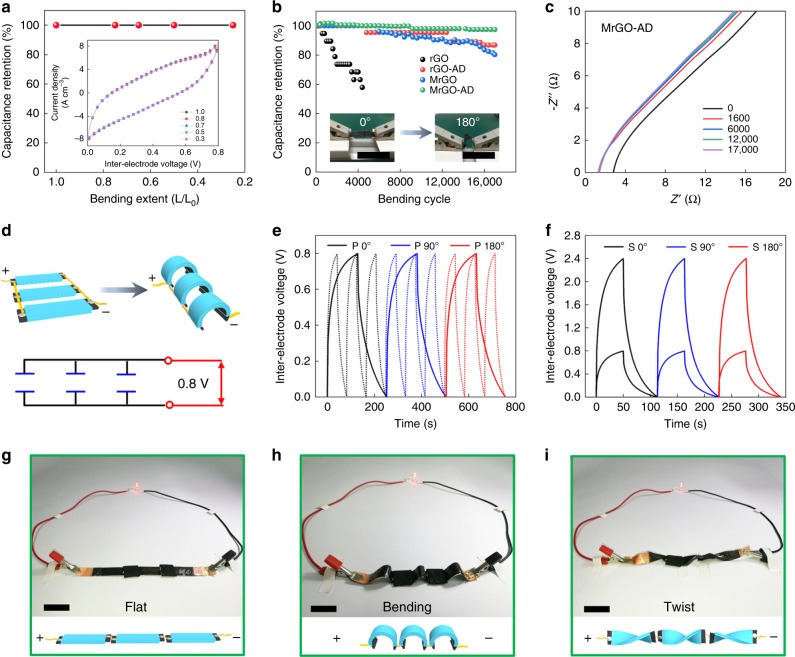
The effect of severe bending of a supercapacitor. **a** Capacitance retention as a function of the bending extent of the supercapacitor (which is defined as the ratio of the end-to-end separation in the bent supercapacitor to this separation for the non-bent supercapacitor). **b** Capacitance retention for supercapacitors based on rGO, rGO-AD, MrGO, and MrGO-AD sheets during up to 17,000 bending cycles to a bending extent of 25% (which corresponds to a 180° angle between supercapacitor ends). Scale bar, 2 cm (inset). **c** Nyquist plots for a MrGO-AD supercapacitor, which were measured after various cycles of bending to the 180° angle of b. **d** Schematic diagram of three in-parallel MrGO-AD supercapacitors (both unbent and with 180° angle bending) and the corresponding circuit diagram. Galvanostatic charge-discharge curves for three **e** in-parallel and **f** in-series MrGO-AD supercapacitors. **g**–**i** In-series operation of three MrGO-AD supercapacitors to light an LED (~1.7 V) when in g, flat. h, 180° bent. **i** in twisted states. Scale bar, 2 cm (**g**–**i**).

In addition to remarkable electrochemical performance, the MrGO-AD supercapacitors also exhibit remarkable electrochemical durability during cycles of extreme bending. Figure 5a shows that this supercapacitor retains ~100% of its capacitance when severely bent (so that the separation between capacitor ends is reduced to 25% of the end-to-end separation for the planar supercapacitor). As shown by the figure inset, this severe bending does not measurably effect the CV curve for the supercapacitor. Figure 5b shows the effect of this 25% bending (which provides a 180° angle between capacitor ends) during 17,000 cycles results in only a 2% decrease in capacitance. This figure also shows that the rGO-AD and MrGO supercapacitors lose ~13% of their initial capacitance during such cycling during repeated bending, and that only 4400 cycles of this bending decreases the capacitance of a rGO supercapacitor by 42%. Hence, the electrochemical cyclability during bending increases with the increasing strength and toughness of the sheet. Using electrochemical impedance spectroscopy, the dependence of electrochemical impedance for a MrGO-AD supercapacitor was investigated for up to 17,000 bending cycles to provide the results shown in Fig. 5c. These results show that a significant decrease in electrochemical resistance (from 2.8 to 1.3 Ω) occurs during the first 1600 bending cycles, and the further cycling to a total of 17,000 bending cycles causes on a small resistance increase (from 1.3 to 1.4 Ω).

We assembled three identical MrGO-AD supercapacitors in parallel and three of these supercapacitors in series (Fig. 5d) and characterized their performance during galvanostatic charge-discharge when bent by 0°, 90°, and 180º. The performance was independent of the bending angle. Also, the in-parallel connected supercapacitors enabled the discharge time to be reduced by a factor of three when the same constant current was used for both the reference single supercapacitor and the three supercapacitors (Fig. 5e). Connecting these three supercapacitors in-series (Fig. 5f), and using the same current for charging both a single supercapacitor and the three supercapacitors, provided a three times higher voltage (2.4 V) for the three supercapacitors than for the single supercapacitor (0.8 V). Figure 5g–i, show that the three in-series-connected supercapacitors can brightly light the red LED (which requires ~1.7 V) when flat, bent by 180º, or severely twisted. Supplementary Fig. 32 shows that an array of cantilevered MrGO-AD supercapacitors can light a display screen while this array is stretched by 200%.

## Discussion

This work demonstrates that strong, supertough sheets can be made by a process that involves the reaction of an exfoliated MXene with exfoliated GO during filtration-based sheet fabrications. By reducing the GO and infiltration of a π-π bridging agent for the rGO, high strength, super high toughness, and high electrical conductivity sheets are obtained. These superior properties are shown to result from the synergetic effects of MXene bridging and the π-π bridging, which decrease porosity and increase the alignment of rGO platelets. In situ Raman spectroscopy and molecular dynamics simulations revealed the synergistic interfacial interactions mechanism. Resulting MrGO-AD sheets are exploited as highly flexible supercapacitors that provide a combination of high volumetric energy storage and high volumetric power generation.

## Methods

### Materials

Ti_*3*_AlC_*2*_ powders were purchased from Jilin 11 Technology Co., Ltd. The 1-aminopyrene (AP) and disuccinimidyl suberate (DSS) were purchased from Sigma-Aldrich Co. Ltd. The solution of hydroiodic acid (HI, 57 wt%) was obtained from Macklin Co. Ltd. All materials were used without further purification.

### Preparation of suspensions of GO platelets

Following Hummers’ method^42^, concentrated H_2_SO_4_ (70 mL) was added to a mixture of graphite flakes (2.0 g) and NaNO_3_ (1.2 g). Then the mixture was cooled to 0 °C with stirring for 1 h. Afterwards, KMnO_4_ (6.0 g) was added slowly in portions, keeping the reaction temperature below 10 °C for 1 h. The reaction was stirred for another 4 hour in an ice bath (below 10 °C). When the reaction was finished, the mixed solution was warmed to 35 °C and stirred for 30 min. After water (300 mL) was added slowly, external heating was introduced to maintain the reaction temperature at 98 °C for 15 min. Then the hot solution was poured into 400 mL of water (60 °C). An additional 30% H_2_O_2_ (3 mL) was added and the mixture stood for 2 days. After multiple washings, centrifugations, and ultrasonic dispersion, the suspension of GO platelets was obtained with the concentration of ~5.0 mg mL^−1^.

### Preparation of a solution of monolayer MXene nanosheets

The monolayer MXene nanosheets were prepared as previously described^9^. Ti_*3*_AlC_2_ was added to 20 mL solution of 9 M HCl that contained 2 g of LiF and the solution was stirred at 35 °C for 24 h. After completely reaction, the MXene product was washed with deionized water for five cycles, which involved 5 min of centrifugation at 3500 rpm for each cycle. This produced a dark-green supernatant solution of MXene nanosheets having a pH of ≈7. After a final separation of the MXene from the solution using the centrifuge, the MXene was added to deionized water and sonicated for 1 h in an ice bath. After centrifugation at 3500 rpm for 1 h, the obtained exfoliated supernatant had a MXene concentration of ~1.0 mg mL^−1^.

### The fabrication of MXene-functionalized graphene sheets

MGO sheets were fabricated by vacuum-assisted filtration. A solution containing the desired relative amounts of dispersed monolayer MXene and GO nanosheets was sonicated for 10 min. Afterwards, the solution was stirred for 6 h until reaction between the MXene and GO nanosheets was completed. Then the MGO sheets were obtained by vacuum-assisted filtration, and dried in a vacuum at 60 °C for 12 h. The MGO sheets were reduced by HI for 6 h, and then the sheets were immersed in ethanol at room temperature to remove unreacted HI. Six types of reduced MGO sheets were prepared by adjusting the weight ratio of monolayer MXene to GO platelets, and labelled MrGO-I, MrGO-II, MrGO-III, MrGO-IV, and MrGO-V. Neat GO and rGO sheets were obtained by using same method. Separate DMF solutions 24 mM of 1-aminopyrene (AP) and disuccinimidyl suberate (DSS) were stirred for 2 h. Then the long-chain AD molecules were formed when these two solutions were mixed at a molar ratio of 1:1 and stirred for 12 h. MrGO-AD sheets were obtained when MrGO sheets were immersed in the AD solution for 2 h, followed by washing six times with DMF and then six times with ethanol. The rGO-AD and MGO-AD sheets were prepared is an analogous manner.

### Fabrication of the flexible supercapacitors

The PVA solution was first prepared through dissolving PVA (*M* = 88,000–90,000) in water with continuous stirring at 90 °C until completely dissolved (6 g PVA/60 mL H_2_O). Afterwards, the transparent PVA/H_3_PO_4_ gel electrolyte was obtained by adding 6.0 g H_3_PO_4_ into the above solution and stirring for another 12 hours. Then, two identical sheets were immersed in the gel electrolyte for 4 h (leaving 0.5 cm length at the end of the sheets uncoated) and then slowly removed. Two pieces of dried and gelled electrodes were placed in parallel to assemble flexible supercapacitors. The end of each electrode was attached to a strip of Cu using silver paste to provide electrical connections.

### Electrochemical measurements

All electrochemical measurements were made at room temperature on an electrochemical workstation (CHI 660e). Cyclic voltammetry (CV) measurements on the supercapacitors were conducted at scan rates of 10 mV s^−1^ to 1000 mV s^−1^. Galvanostatic charge-discharge (GCD) curves were conducted at volumetric current densities of from 1.0 to 8.6 A cm^−3^. Galvanostatic measurements of stability during cycling were conducted using a current density of 8.0 A cm^−3^. Electrochemical impedance spectroscopy (ESR) measurements on the supercapacitors were obtained from 10 mHz to 100 kHz. CV measurements on the effects of bending on supercapacitors performance were made using a scan rate of 50 mV s^−1^. Gravimetric specific capacitances of supercapacitor electrodes were calculated from CV curves (C_s,g1_) and from GCD curves (C_s,g2_). These gravimetric capacitances, the volumetric capacitance of a single supercapacitor electrode (C_s,v2_), and the volumetric capacitance of entire device (C_w,v2_) were obtained as follows:

1 $${\mathrm{Cs,g1}} = \frac{{2{\smallint}{\mathrm{IdV}}}}{{\Delta Vm_{\mathrm{s}}}}$$

2 $${\mathrm{Cs,g2}} = \frac{{{\mathrm{2I}}\Delta {\mathrm{t}}}}{{{\mathrm{m}}_{\mathrm{s}}{\mathrm{(}}\Delta {\mathrm{V}} - {\mathrm{IR)}}}}$$

3 $${\mathrm{C}}_{{\mathrm{s,v2}}} = \frac{{{\mathrm{C}}_{{\mathrm{s,g2}}}{\mathrm{m}}_{\mathrm{s}}}}{{V_{\mathrm{s}}}}$$

4 $${\mathrm{Cw,v2}} = \frac{{{\mathrm{C}}_{{\mathrm{s,g2}}}V_{\mathrm{s}}}}{{2V_{\mathrm{w}}}}$$

where *I* is the current (A), *V* is the potential (V), ∆V is the potential window (V), m_s_ is the loading mass of single electrode, ∆*t* is the discharge time (s), IR is the voltage drop (V), *V*_s_ is the volume of the single electrode (cm^−3^), and *V*_w_ is the volume of the whole device (cm^−3^).

The volumetric energy density of the entire supercapacitor (*E*_w_) (mWh cm^−3^) was calculated using Eq. (5). The volumetric power density of the supercapacitor (*P*_w_) (mW cm^−3^) was calculated from galvanostatic curves at different charge/discharge current densities using Eq. (6).

5 $$E_{\mathrm{w}} = \frac{{{\mathrm{C}}_{{\mathrm{w,v2}}}\Delta V^{\mathrm{2}}}}{{{\mathrm{2}}\;\times\;{\mathrm{3600}}}}$$

6 $$P_{\mathrm{w}} = \frac{{E_{\mathrm{w}}\;\times\;{\mathrm{3600}}}}{t}$$

### Molecular dynamics simulations

To reveal the fracture mechanism of MrO-AD sheet, molecular dynamics (MD) simulations were performed using the large-scale atomic/molecular massively parallel simulator package (LAMMPS).^1^ In our MD simulations, the epoxy and hydroxyl functional groups are considered in the rGO sheet, and the ratio is *n*_-O-_:*n*_OH_:*n*_C_ = 0.03:0.03:1.0, which is close to the experimental sample. The width of single rGO and MXene is 2.74 nm, while the length for rGO and MXene is ~22.30 and ~3.51 nm, respectively. The hybrid sheet is constructed by depositing rGO flakes in a brick-wall manner with an initial interlayer distance of 0.55 nm, while an MXene stacking with three sheets and four AD molecules is intercalated into the rGO platelets as shown in Supplementary Fig. 25. The force field for the rGO sheet and AD molecule is all-atom optimized potentials for liquid simulation (AA-OPLS). The particle-particle-particle mesh (PPPM)^2^ method was used to include the long-range Columbia interaction, while the 12-6 Lennard-Jones potential was used to describe the non-bonded van der Waals interaction. The MXene layer is viewed as a rigid layer to explore the sliding behavior under external stress. The periodic boundary conditions along three directions were used in the MD simulations to avoid the size effect of the simulated cell. The initial-prepared systems were relaxed in the NPT ensemble with a temperature of 300 K and a pressure of 1 bar for 500 ps, which are controlled by the Berendsen thermostat and barostat.^3^ The time step was 0.5 fs for the MD simulation. After the system was fully relaxed, the composite sheet were stretched in the *X* direction with an engineering strain rate of 1 × 10^7^ s^−1^ while the pressure along *Y* and *Z* direction was kept at ~1 bar. During the stretching process, the corresponding stress, strain, and atomic structure were recorded to obtain the mechanical and structural response of MrGO-AD sheet.

### First-principle calculations

The interfacial energy of MXene/MXene, MXene/rGO, and rGO/rGO interfaces were explored by performing first-principles calculations using a plane-wave basis set method based on the density functional theory. For the core-valence electron interaction, ultra-soft pseudopotentials^4^ are used, and the Perdew-Burke-Ernzerhof parameterization^5,6^ of the generalized gradient approximation is adopted for the exchange-correlation functional. To account for the dispersion interaction, the vdW-D3 is invoked as a non-local correlation functional in all the calculations^7^. All the calculations were performed using the Vienna ab initio Simulation Package (VASP)^8^. For all results presented, an energy cutoff of 500 eV is used for the plane-wave basis set. A 1 × 1 × 1 Monkhorst-Pack mesh grid for sampling *k* points is set up for Brillouin zone integration. These settings are verified by achieving a total energy convergence of <1 meV/atom. Geometry relaxation is carried out before studying the structural properties and total energy, where the force-on-atom is converged below a threshold of 0.01 eVÅ^−1^. The interfacial energy is defined as *Γ* = (*E*_AB_ − *E*_A_ − *E*_B_)/*S*, where *E*_AB_ is the total energy of rGO/rGO, MXene/MXene, or MXene/rGO systems, and *E*_A_ and *E*_B_ are the energy for single-layer rGO or MXene, respectively.

### Characterization

High-resolution transmission electron microscope (HR-TEM) images were obtained using a FEI Tecnai G2 F30 instrument and an acceleration voltage of 300 kV. AFM images were obtained by using a Bruker Dimension Icon. SEM images were collected using a SU8010 scanning electron microscope at a voltage of 5 kV. Zeta potentials were obtained using a Zen3600 Zetasizer Nano ZS. TGA results were obtained under nitrogen using a Q600 SDT from Thermal Instruments and a heating rate of 10 K min^−1^ between 35 and 800 °C. XRD patterns were recorded using CuKα radiation and a D8 ADVANCE X-ray diffractometer from Bruker. FTIR spectra were obtained at room temperature by using a Nicolet 6700 spectrometer. XPS spectra were obtained using a Thermo Scientific ESCALAB 250 Xi with a monochromatic Al-Kα X-ray source. UV-vis spectra were recorded by a SHIMADZU UV-3600. In situ Raman spectra were measured using an apparatus from Renishaw Laser using a laser excitation of 632 nm. Electrical conductivities were measured using a standard two-probe method and a Keithley 2400 source meter. WAXS and SAXS measurements were conducted on a XEUSS WAXS/SAXS system from Xenocs, which was equipped with a rotating anode X-ray source (operated at 50 kV and 0.6 mA). These measurements used a Pilatus 300 K detector. The WAXS detector was placed 125 mm from the samples, while the SAXS detector was placed 2316 mm from the samples. Tensile stress–strain curves at room temperature were measured on 2-cm-long, 3-mm-wide sheets using a SUNS UTM4000 Tester at a loading rate of 1 mm min^−1^. The thickness of sheet strips was measured by SEM. The results for each sheet type were evaluated from the average value of at least three samples.

## Supplementary information

Supplementary Information

## Data Availability

The data that support the findings of this study are available from the corresponding author upon reasonable request.
