# Supplementary material for: Super-tough MXene-functionalized graphene sheets
Source: Nat Commun. 2020 Apr 29;11:2077. doi: 10.1038/s41467-020-15991-6 (PMC7190721; doi:10.1038/s41467-020-15991-6)
Supplement: Supplementary file 1 — Supplementary Information [file 41467_2020_15991_MOESM1_ESM.pdf]

Supplementary Information

Super-Tough MXene-Functionalized Graphene Sheets

Zhou et al.

Supplementary Figures

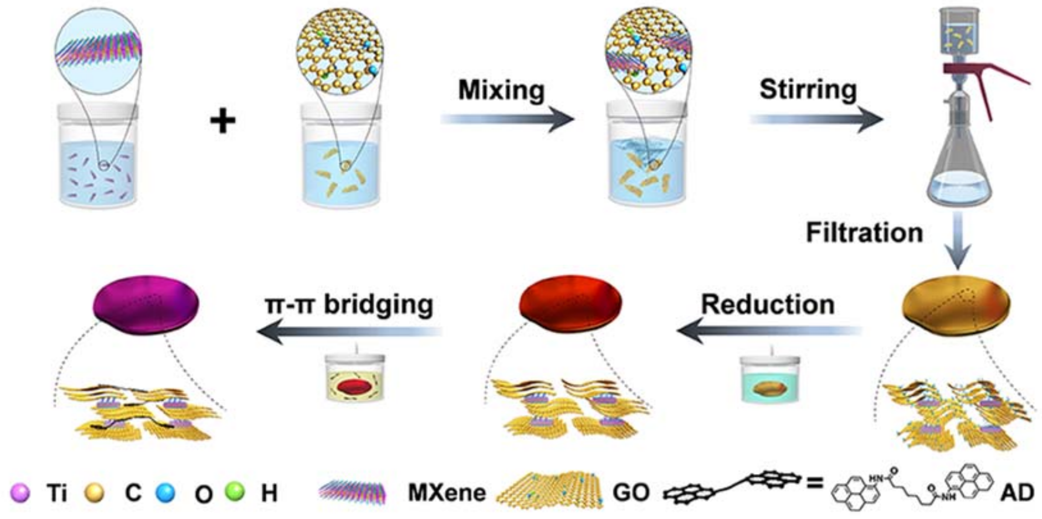

Supplementary Figure 1 | Illustration of the process for fabricating sheets. The manufacturing process of MrGO-AD film with stirring, vacuum filtration, chemical reduction, and chemical cross-linking with the long-chain AD molecules via π-π bridging interaction.

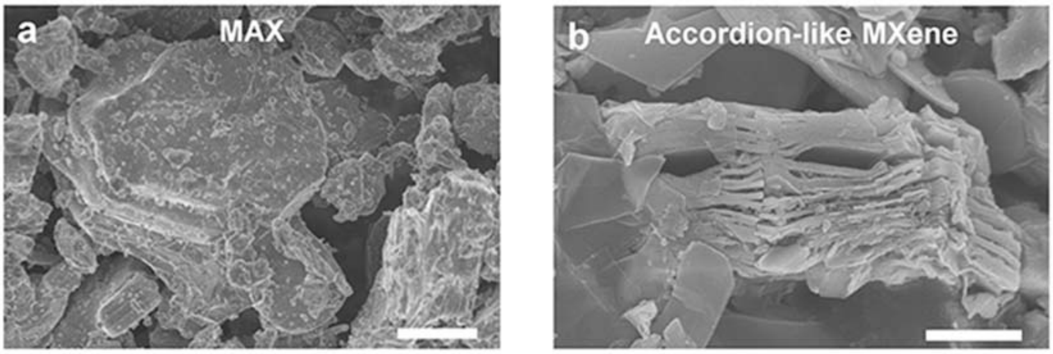

Supplementary Figure 2 | SEM images of **a** Ti_3AlC_2 and **b** accordion-like MXene ($Ti_3C_2T_x$). Scale bar, 5 μm (a) and 2 μm (b).

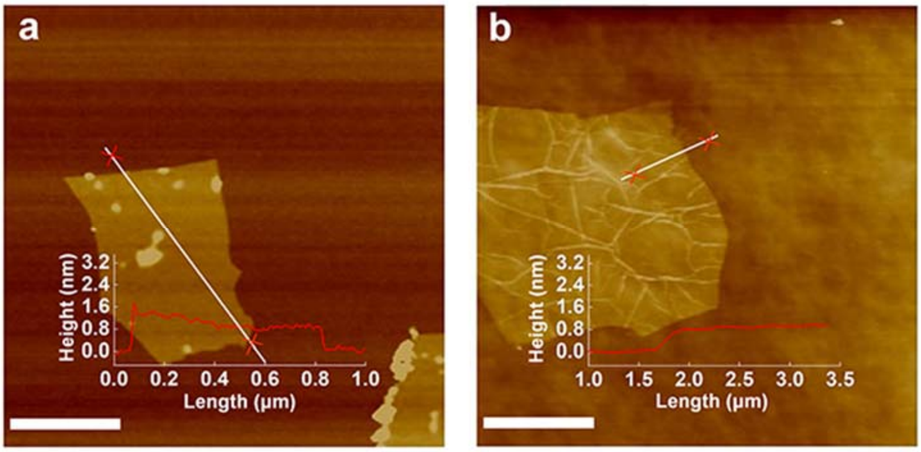

Supplementary Figure 3 | AFM images of **a** exfoliated MXene nanosheets and **b** GO platelets, which show that the thicknesses of the MXene nanosheets and the GO platelets are ~1.5 nm and ~1.0 nm, respectively. Scale bar, 500 nm (a) and 5 μm (b).

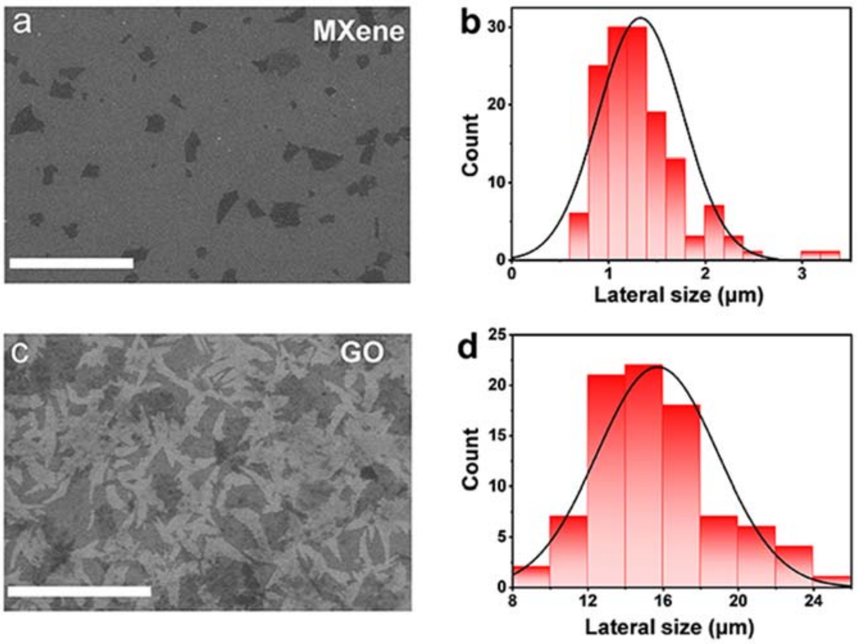

Supplementary Figure 4 | a and **b** SEM image and the size distribution for exfoliated MXene nanosheets. **c** and **d** SEM image and the size distribution for GO platelets. These results show that the thicknesses of the MXene and GO nanosheets are ~1.5 μm and ~16.0 μm, respectively. Scale bar, 5 μm (a) and 50 μm (c).

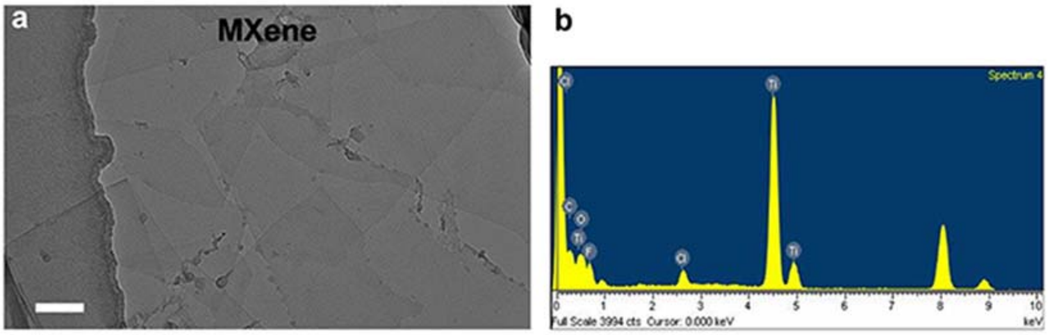

Supplementary Figure 5 | a TEM image of an exfoliated MXene nanosheet and **b** corresponding energy-dispersive spectroscopy (EDS) spectra. The TEM image shows that the MXene nanosheets are monolayer. Scale bar, 100 nm (a).

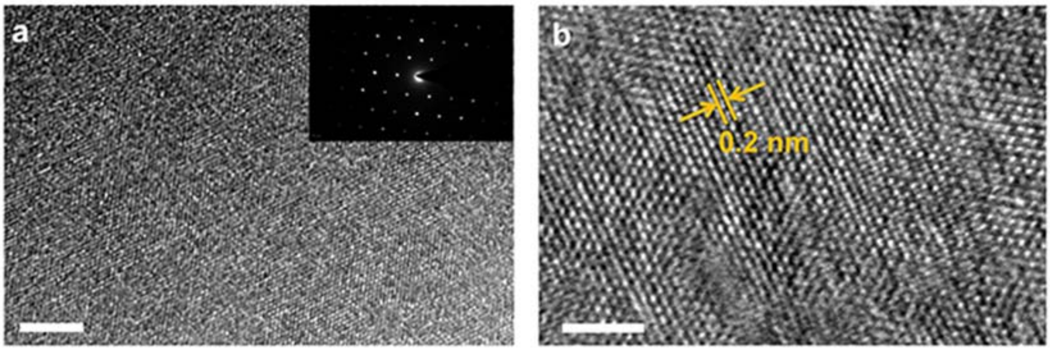

Supplementary Figure 6 | a TEM image of exfoliated MXene nanosheets and **b** corresponding HR-TEM image showing a lattice spacing of 0.2 nm. The selected area electron diffraction pattern (inset in a) shows that the MXene nanosheets are hexagonal single crystals that do not contain obvious defects. Scale bar, 4 nm (a) and 2 nm (b).

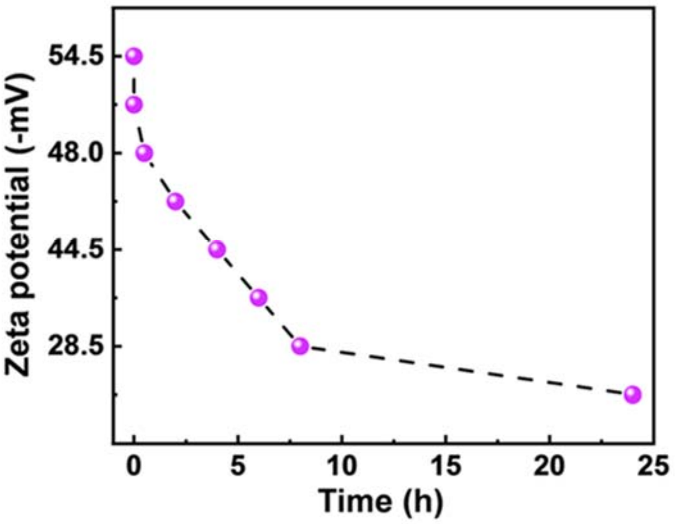

Supplementary Figure 7 | The time dependence of the Zeta potential during the reaction of MXene (M) with GO to form MGO.

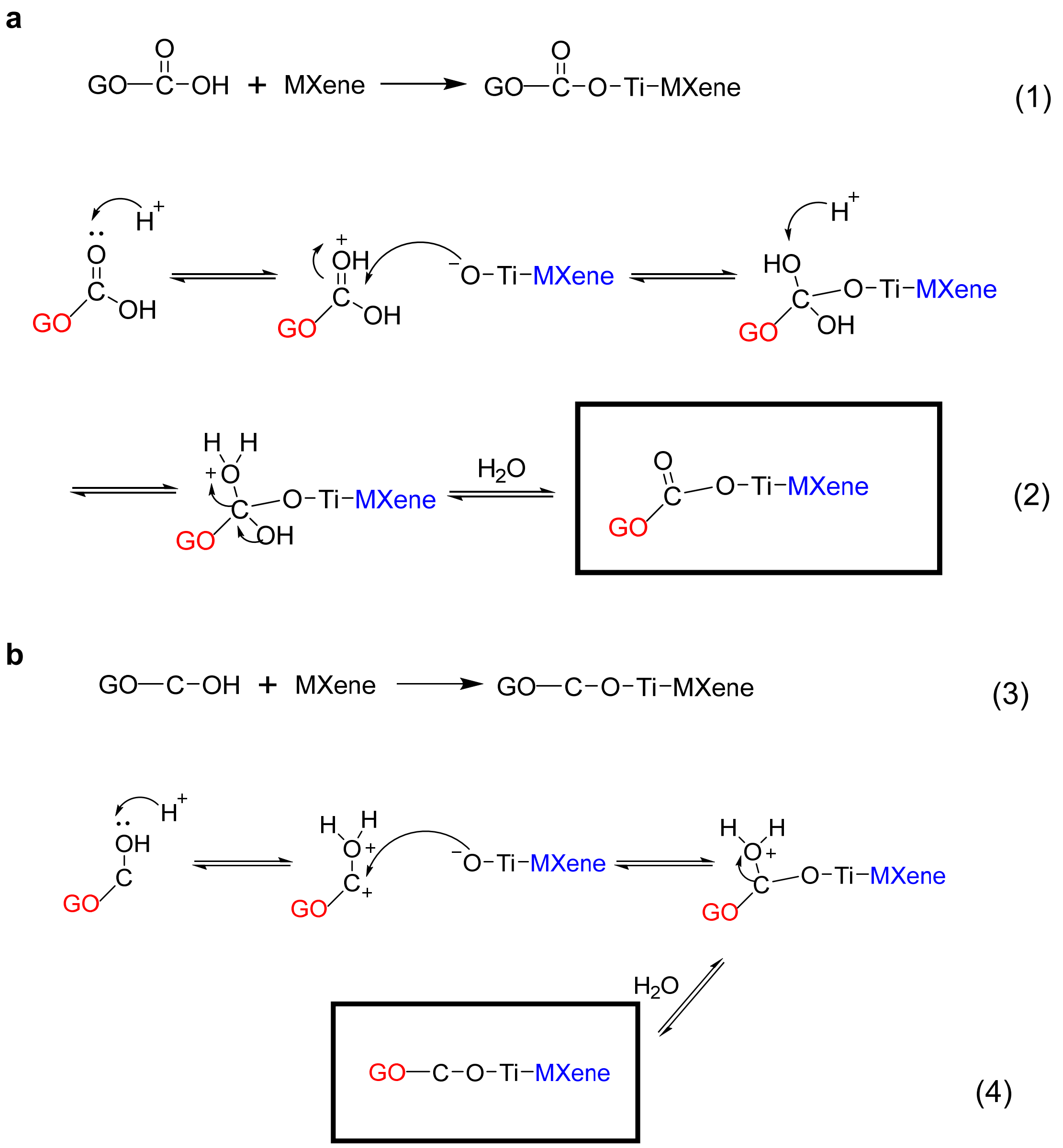

Supplementary Figure 8 | Possible mechanism for the formation of Ti-O-C covalent bonding between MXene nanosheets and GO platelets. **a** In this mechanism, the carboxyl group (-COOH) of a GO platelet reacts with a MXene nanosheet to form Ti-O-C covalent bonding via nucleophilic substitution and dehydration reaction of Supplementary equations (1) and (2). **b** For the mechanism of reaction of Supplementary equations (3) and (4), the hydroxyl group (-OH) of a GO platelet reacts with a MXene nanosheet through nucleophilic substitution and dehydration reaction, producing interfacial Ti-O-C covalent bonding.

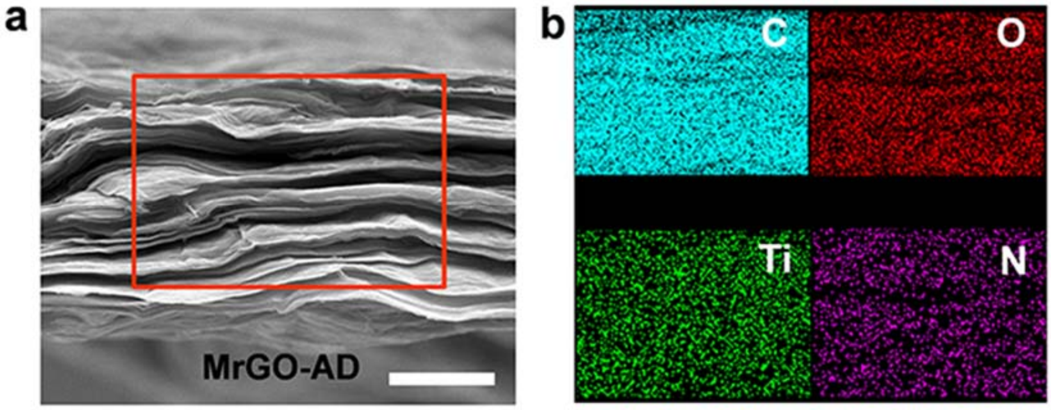

Supplementary Figure 9 | a SEM image of the cross-section of a MrGO-AD sheet. **b** The corresponding EDS map, which shows that Ti and N are uniformly distributed within the volume of the MrGO-AD. The results suggest that the MXene nanosheets and AD molecules have been introduced within the layers of rGO platelets. Scale bar, 2 μm (a).

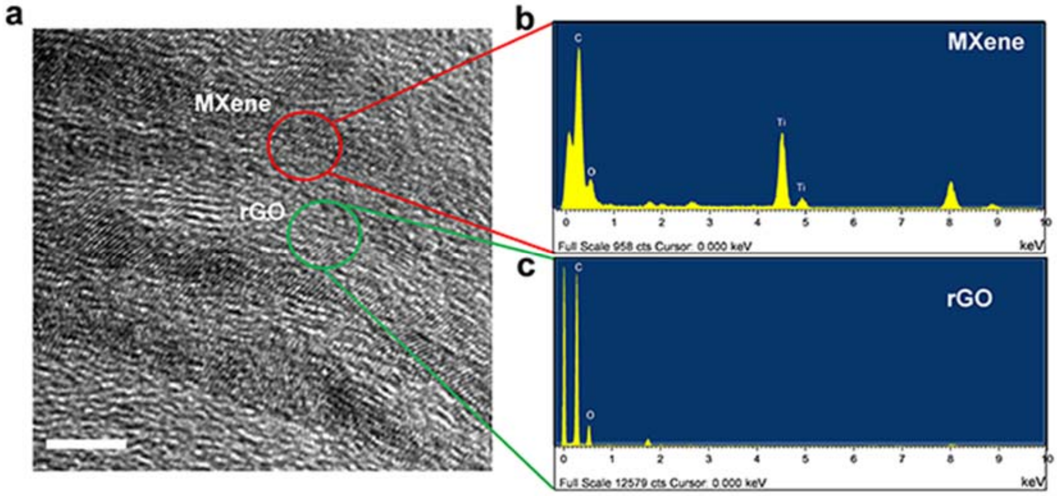

Supplementary Figure 10 | a HR-TEM image of a MrGO-AD sheet. The corresponding EDS spectra of **b** a selected area containing MXene nanosheets and **c** a selected area containing principally rGO platelets. Scale bar, 5 nm (a).

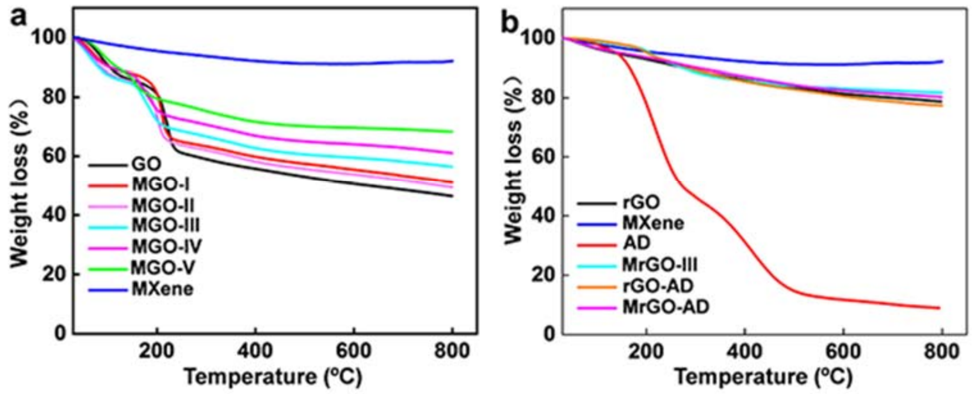

Supplementary Figure 11 | a TGA curves of pure GO, MXene, MGO-I, MGO-II, MGO-III, MGO-IV, MGO-V, and MGO-VI sheets. **b** TGA curves of pure rGO, AD, MrGO-III, rGO-AD-III, and MrGO-AD sheets. These results were obtained in a nitrogen atmosphere using a heating rate of 10 K min^{-1}. The weight ratio of AD (W_2) in the MrGO-AD was 2.0 wt%, according to Supplementary equation (6) (See Supplementary Note 1).

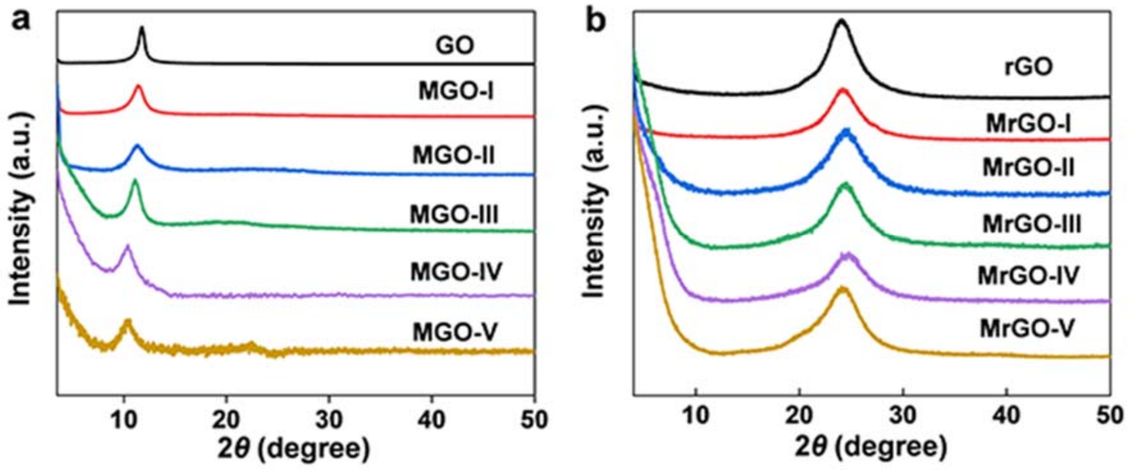

Supplementary Figure 12 | a XRD patterns of MGO sheets. **b** XRD patterns of MrGO sheets. These results show that MXene nanosheets are introduced between GO platelets layers, thereby providing a strain that increases the d-spacing of the GO platelets.

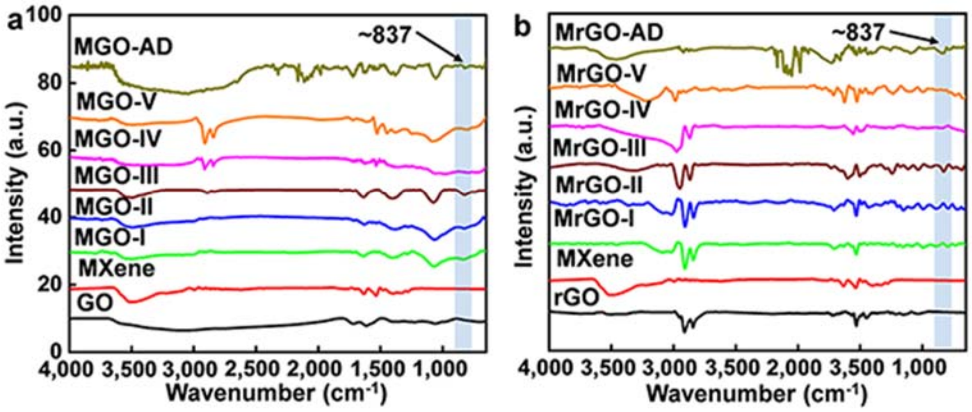

Supplementary Figure 13 | FTIR spectra of **a** MGO sheets and **b** MrGO sheets. The new peak at ~837 cm^{-1} indicates the formation of Ti-O-C covalent bonding between GO and MXene nanosheets.

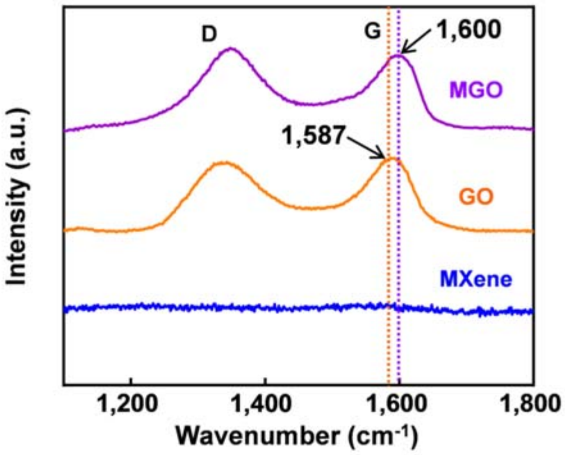

Supplementary Figure 14 | Raman spectra of MXene, pure GO, and MGO sheets, which shows a 13 cm^{-1} upshift of the G-band peak in MGO with respect to the G-band of pure GO. The shift indicates the formation of Ti-O-C covalent bonding between MXene and GO nanosheets.

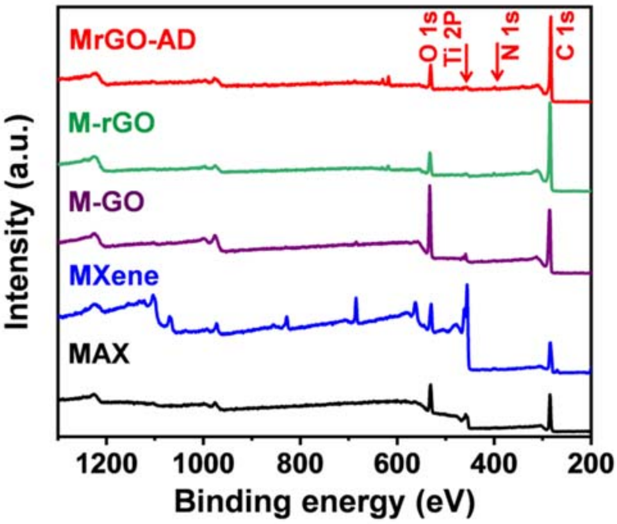

Supplementary Figure 15 | XPS spectra of obtained sheets. The peaks for Ti and N indicate that MXene nanosheets and AD molecules have been introduced into the layers of rGO platelets (via Ti-O-C covalent bonding and π-π bridging interactions).

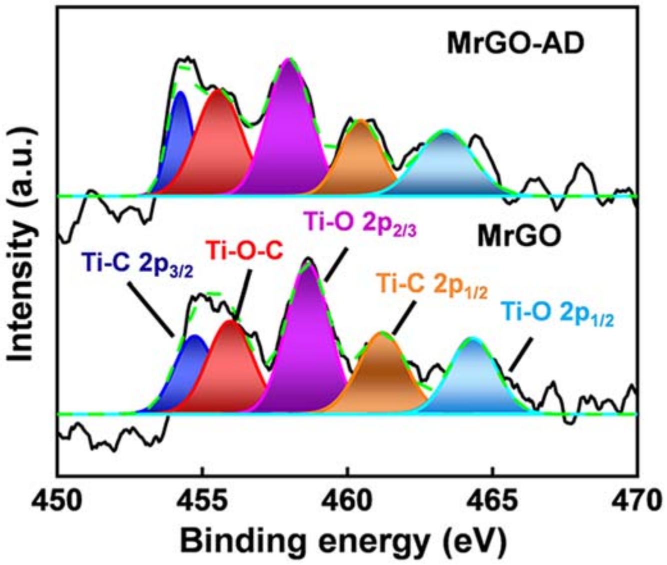

Supplementary Figure 16 | XPS spectra of obtained sheets. Ti 2p spectra of the MrGO and MrGO-AD sheets.

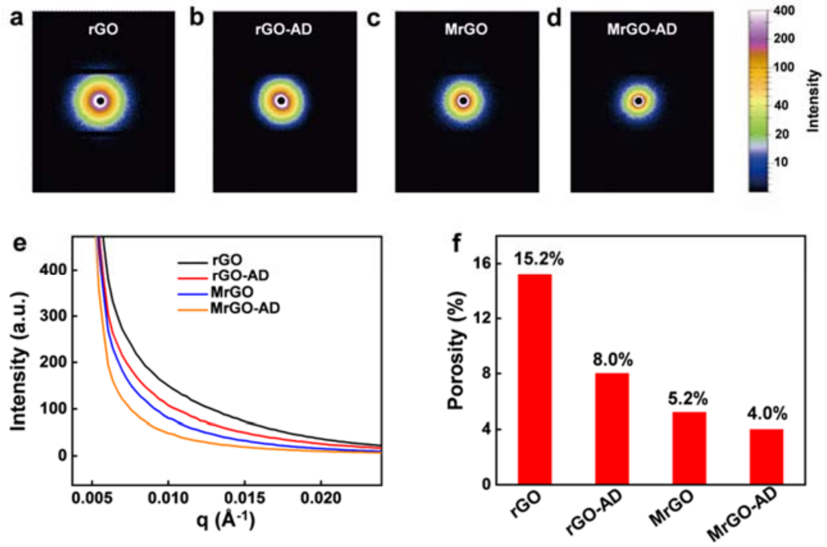

Supplementary Figure 17 | SAXS patterns of **a** pure rGO. **b** rGO-AD. **c** MrGO.; **d** MrGO-AD. **e** The corresponding curves of scattering intensity for the sheets in a. These results (i.e., reduced small angle scattering) show that the AD molecules and MXene make the sheets less porous. **f** A bar graph showing the porosity of the different sheets. The porosity of MrGO and MrGO-AD films decrease to 5.2% and 4.0%, respectively, which is attributed to the filling of microvoids between rGO platelets by MXene nanosheets.

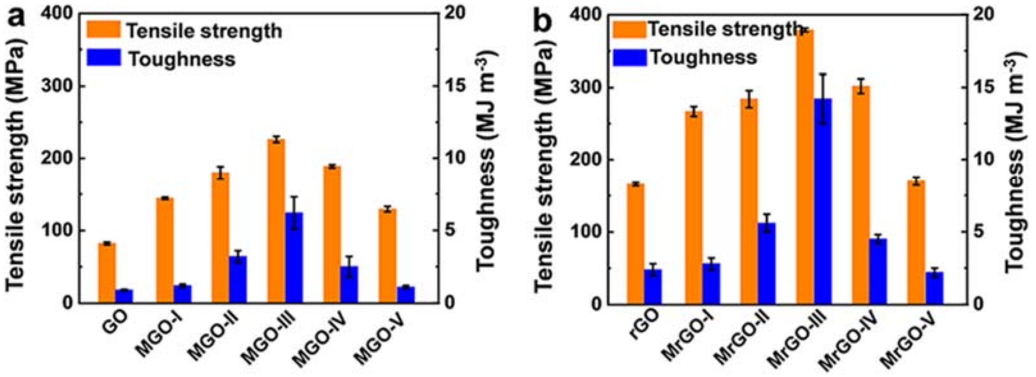

Supplementary Figure 18 | The tensile strengths and toughnesses of **a** MGO sheets and **b** MrGO sheets containing various weight ratios of MXene nanosheets. These results show that MGO-III and MrGO-III sheets, which contain 17.7 wt% MXene, have maximum tensile strengths and toughnesses.

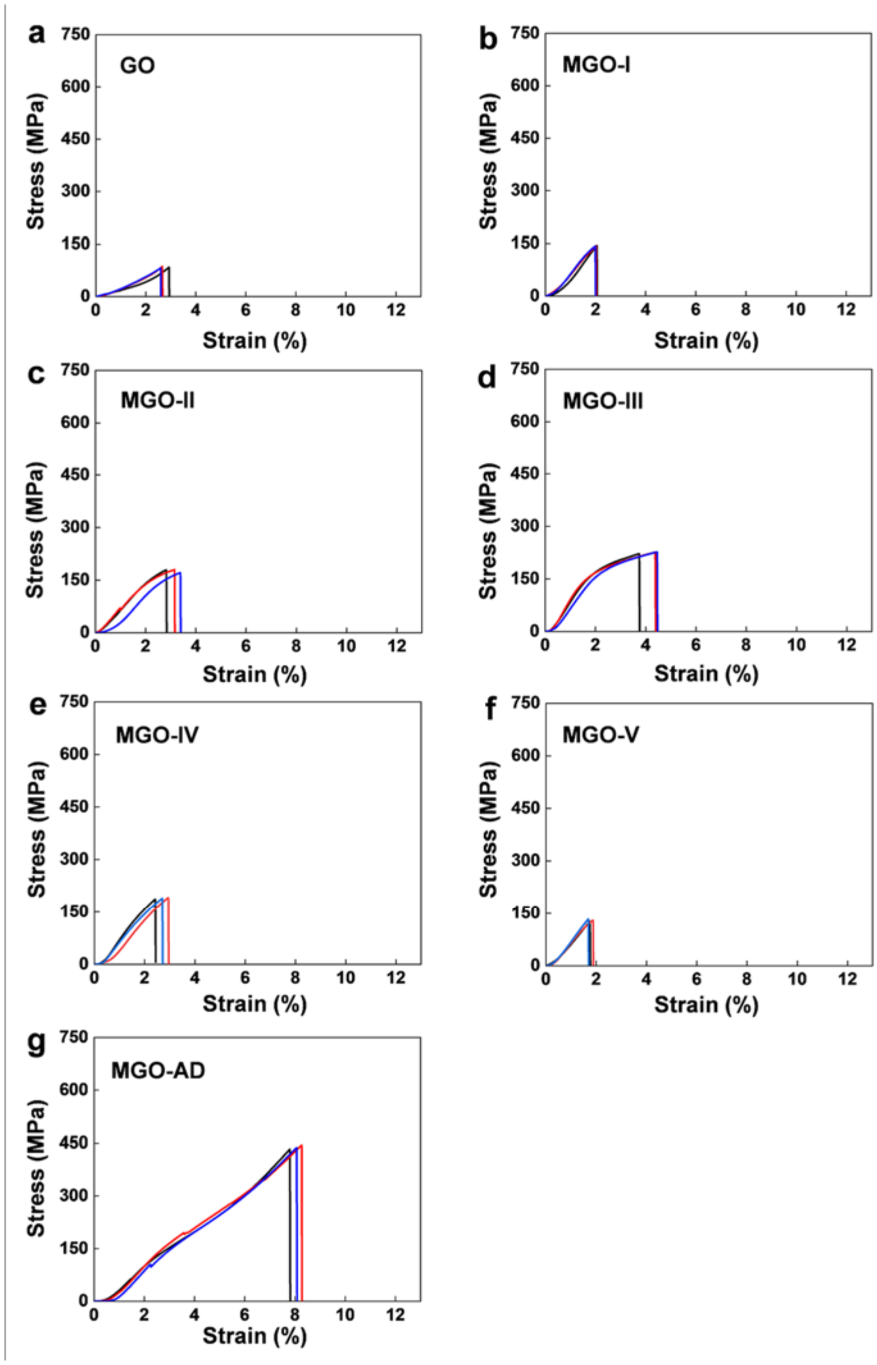

Supplementary Figure 19 | Stress-strain curves for sheets of **a** GO. **b** MGO-I. **c** MGO-II. **d** MGO-III. **e** MGO-IV. **f** MGO-V. **g** MGO-AD.

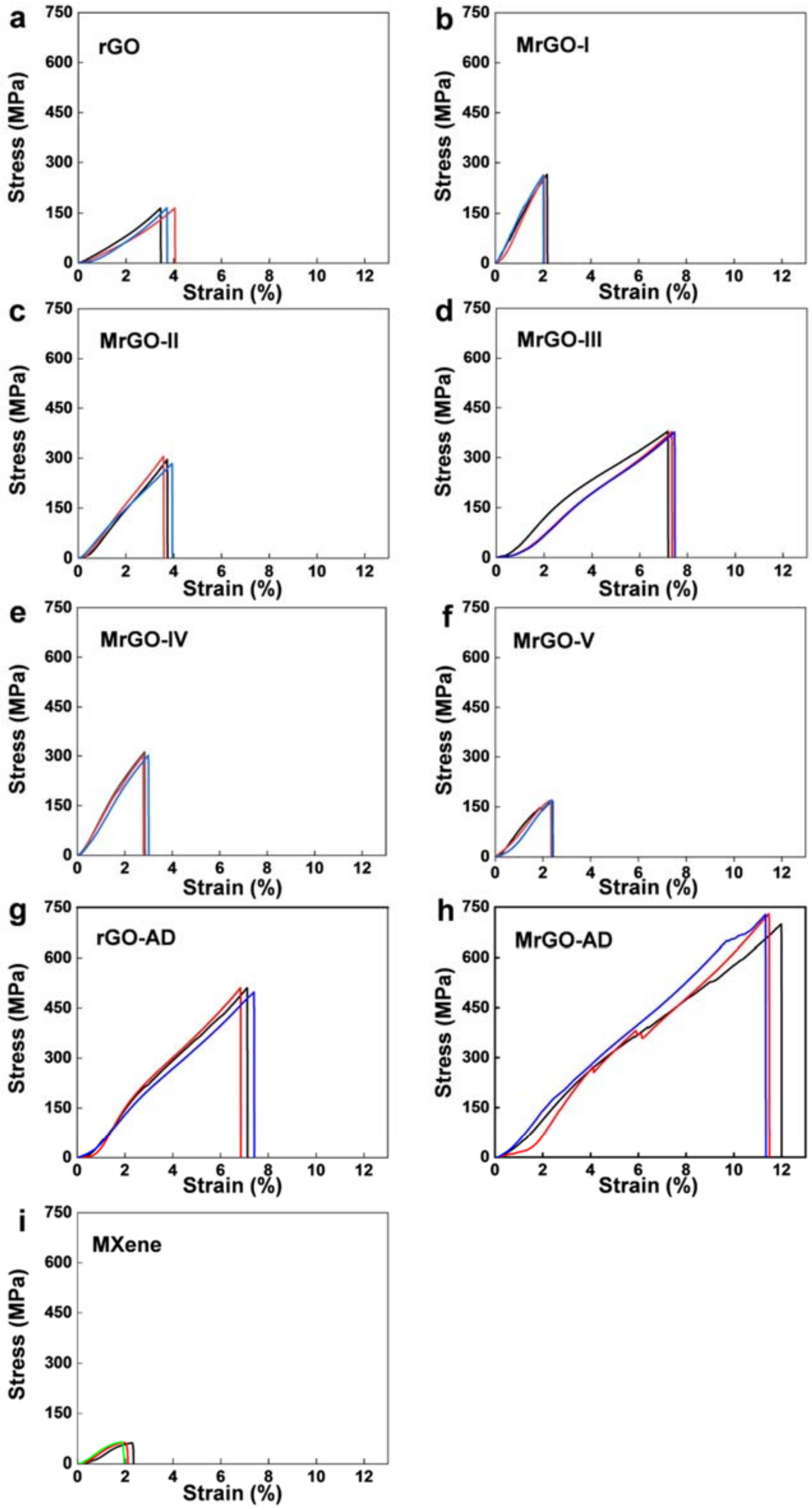

Supplementary Figure 20 | Stress-strain curves for sheets of **a** rGO. **b** MrGO-I. **c** MrGO-II. **d** MrGO-III. **e** MrGO-IV. **f** MrGO-V. **g** MrGO-AD. **i** MXene.

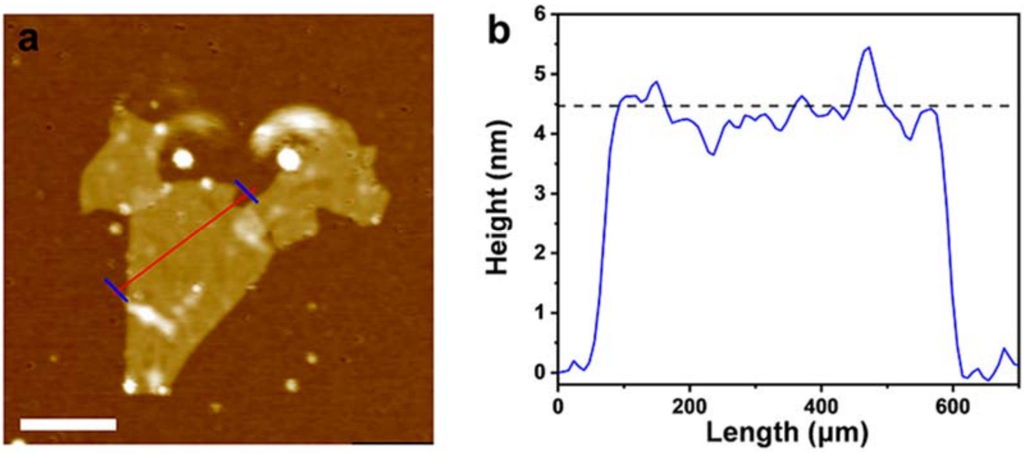

Supplementary Figure 21 | a AFM image of multilayer MXene crystal. **b** The height profile of multilayer MXene crystal showing that the thickness of multilayer MXene nanosheets is ~4.5 nm (~3 layers). Scale bar, 400 nm (a).

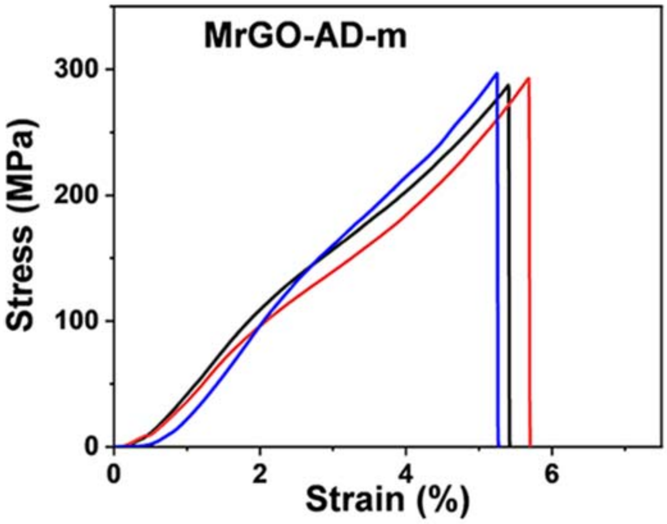

Supplementary Figure 22 | Typical stress-strain curves of the MrGO-AD-m sheet.

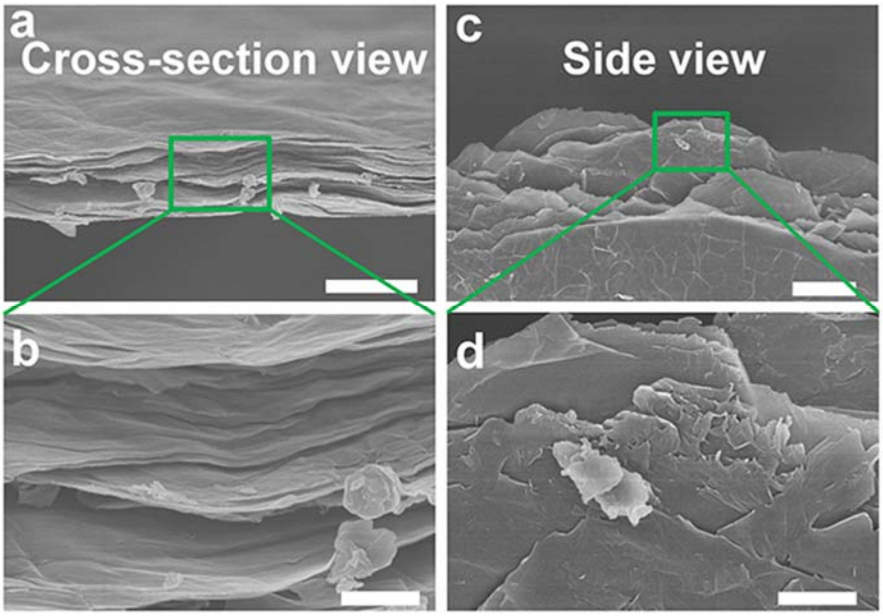

Supplementary Figure 23 | SEM image of the cross-section view (**a, b**) and the side view (**c, d**) of the fracture surface of MrGO-AD-m sheet. Scale bar, 5 μm (a, c) and 1 μm (b, d).

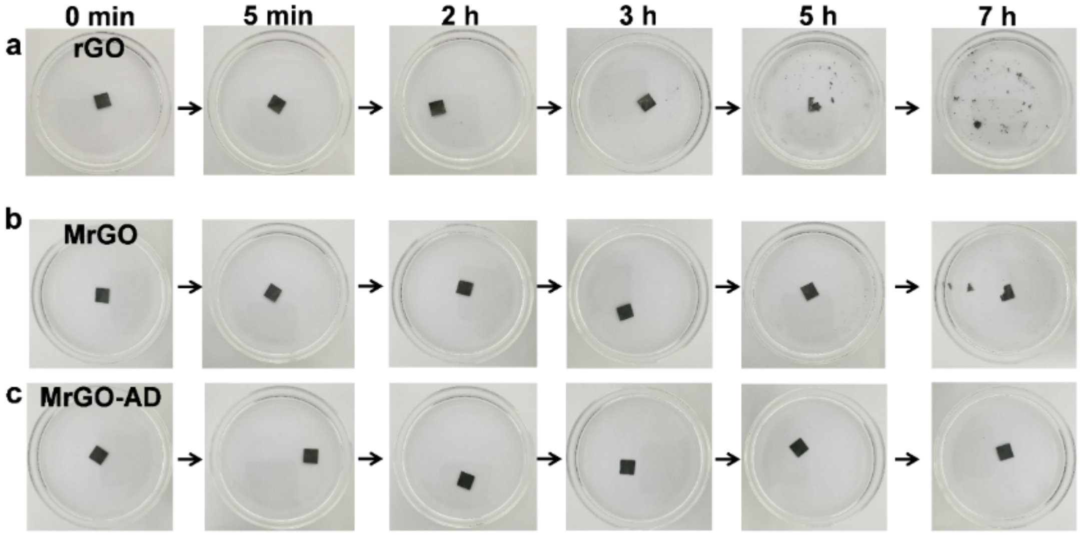

Supplementary Figure 24 | Pictures showing the time dependence of sheet disintegration in ultrasonicated water for **a** rGO. **b** MrGO. **c** MrGO-AD**.** An 100 W, 4.5 KHz ultrasonic source was applied for up to 7 hours. These results show that the neat rGO sheet begins disintegration within 3 hours and completely disintegrates after 7 hours. However, the MrGO and MrGO-AD sheets start to disintegrate after ultrasonic treatment for 5 hours and 7 hours.

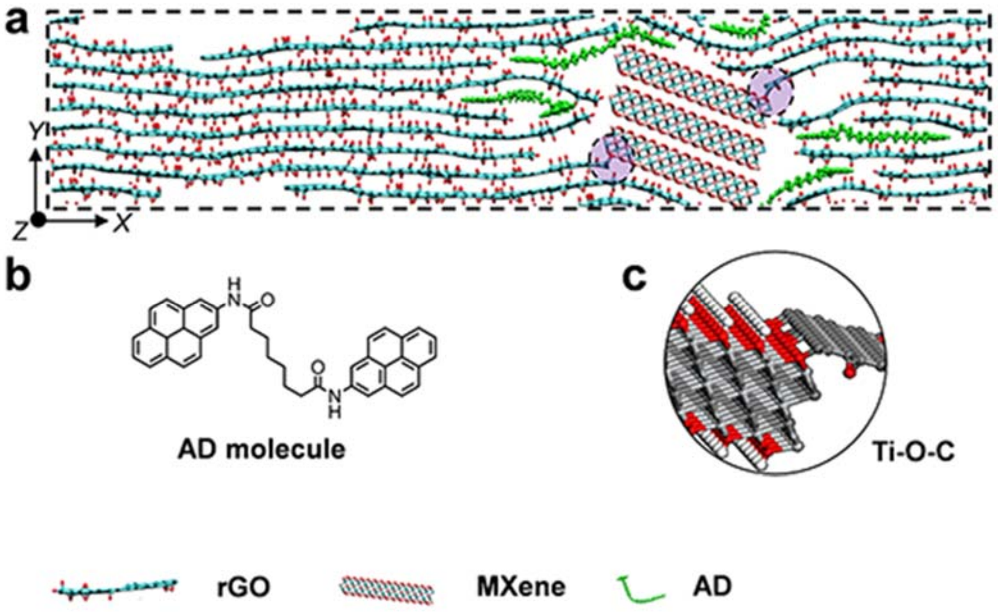

Supplementary Figure 25 | a The relaxed atomic structure of MrGO-AD sheet, where the size of the simulated cell is 22.10×2.74×3.93 nm^3. Cyan, red, white, pink, and green represent carbon atoms, oxygen atoms, hydrogen atoms, titanium atoms, and AD molecules, respectively. The purple regions denote the Ti-O-C covalent bond linking rGO and MXene sheets. **b** and **c** The atomic structure for the AD molecule and detailed Ti-O-C bonds.

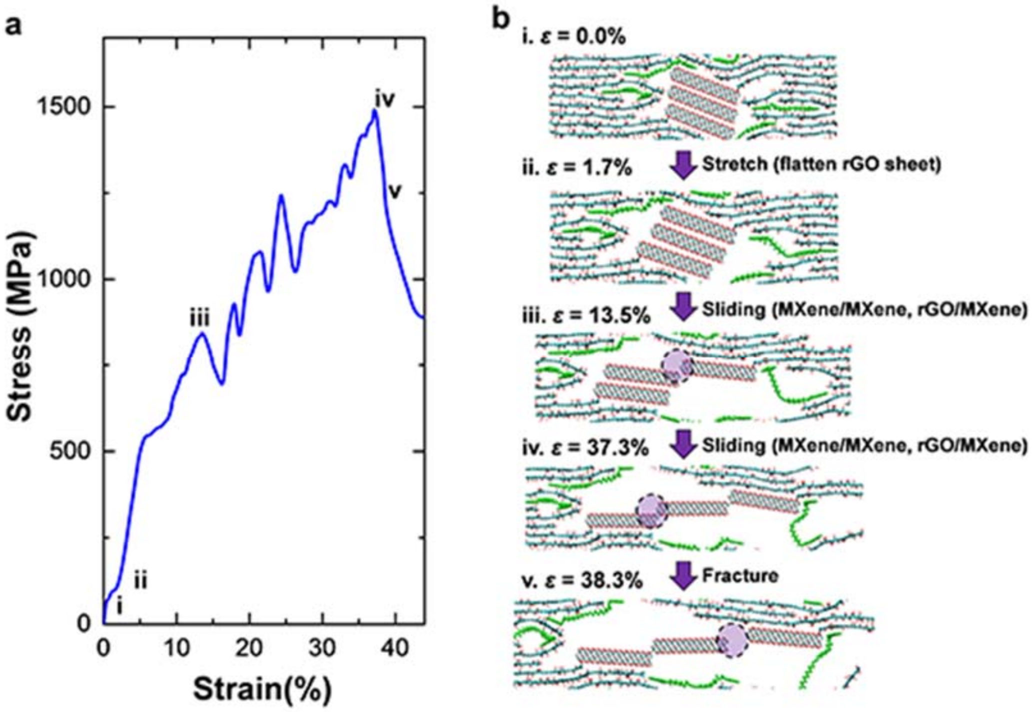

Supplementary Figure 26 | a The stress-strain curves for MrGO-AD sheet from MD simulations. **b** The configuration of rGO, MXene, and AD under different strain, showing the stretching-sliding-fracture mechanism.

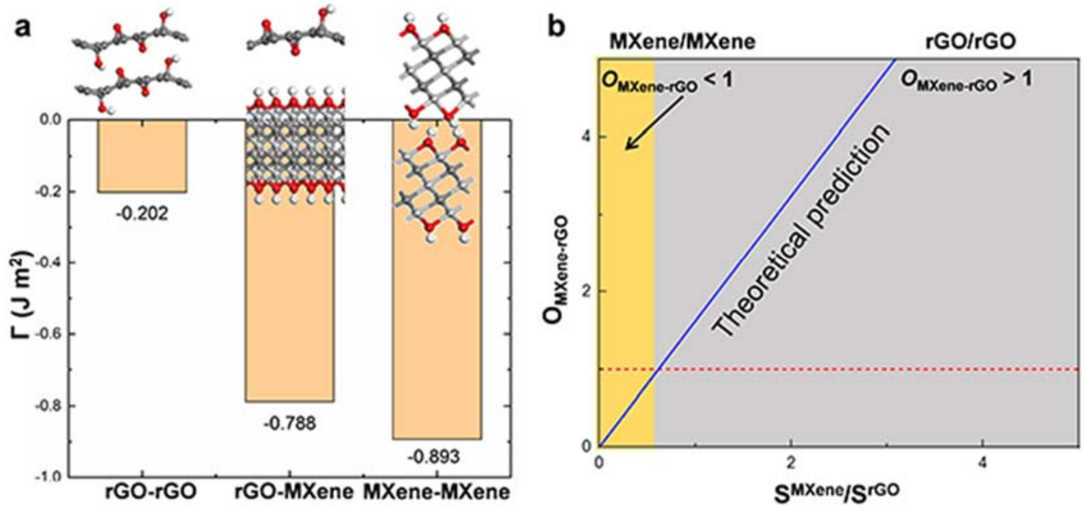

Supplementary Figure 27 | a The interfacial energy for rGO/rGO, rGO/MXene, and MXene/MXene interface calculated from the first-principle calculation. **b** The relation between the sliding order of MXene or rGO and the areal ratio, where S^{MXene} and S^{rGO} represent the surface area of the average flake for MXene and rGO in the hybrid sheet. When $O_{MXene\text{-}rGO} < 1$, the critical stress for MXene slides will be smaller than that of rGO, indicating MXene will slide first. When $O_{MXene\text{-}rGO} > 1$, rGO will instead slide first.

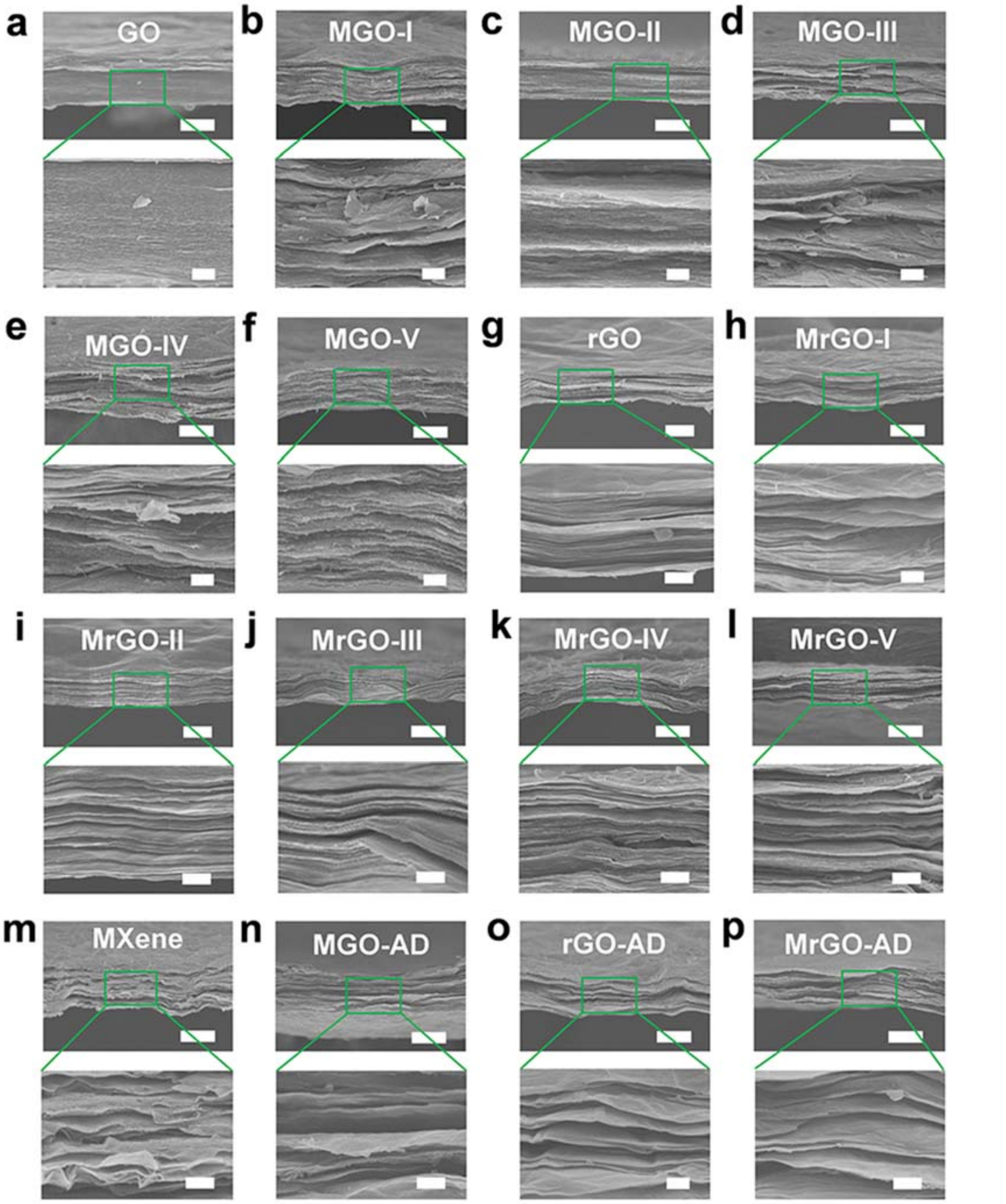

Supplementary Figure 28 | SEM images of the cross-sections of the fracture surface for sheets of **a** neat GO. **b** MGO-I. **c** MGO-II. **d** MGO-III. **e** MGO-IV. **f** MGO-V. **g** neat rGO. **h** MrGO-I. **i** MrGO-II. **j** MrGO-III. **k** MrGO-IV. **l** MrGO-V. **m** MXene. **n** MGO-AD. **o** rGO-AD. **p** MrGO-AD sheets. Scale bar, 5 μm (a-p, above) and 1 μm (a-p, below).

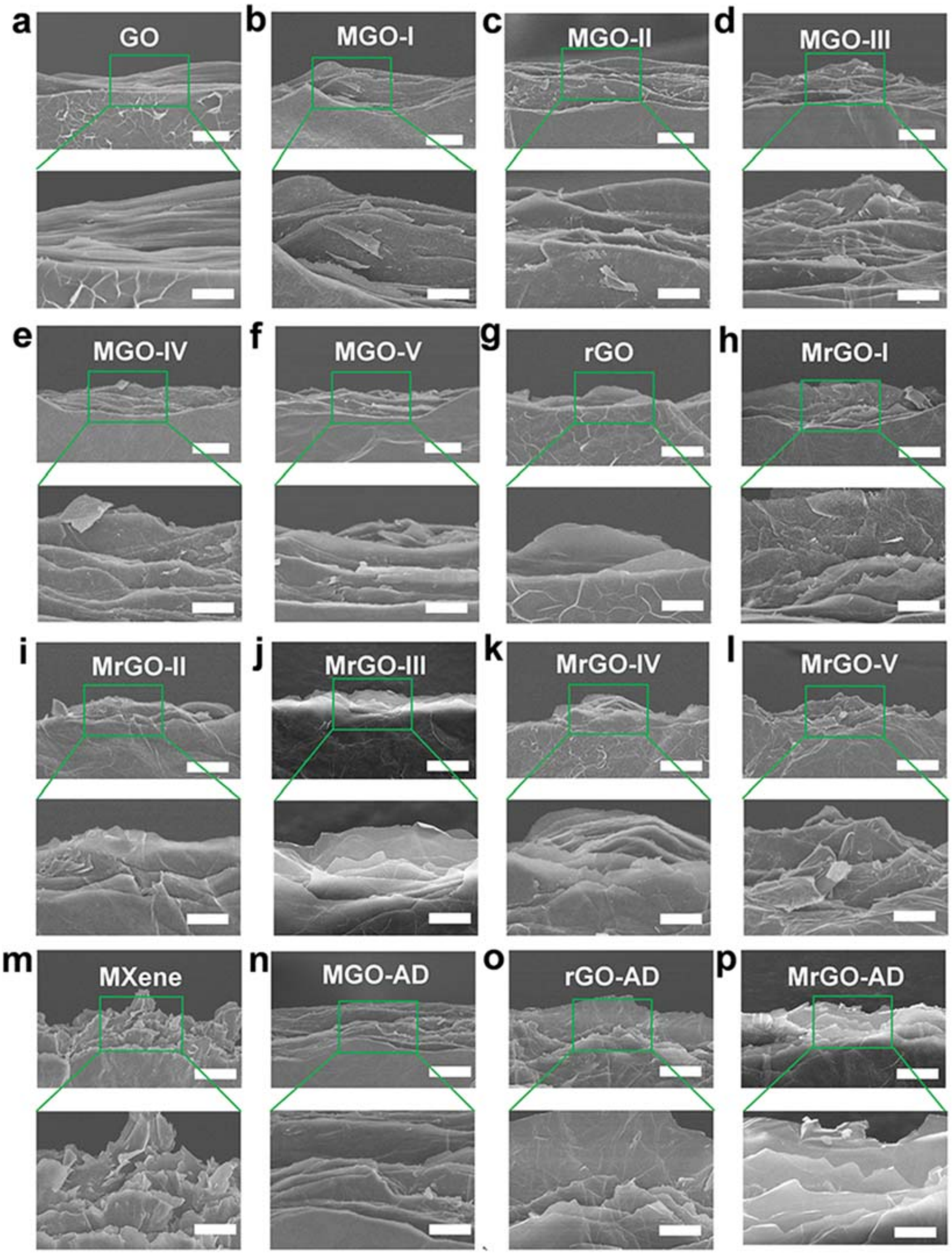

Supplementary Figure 29 | SEM images of the fracture surface of sheets of **a** neat GO. **b** MGO-I. **c** MGO-II. **d** MGO-III. **e** MGO-IV. **f** MGO-V. **g** neat rGO. **h** MrGO-I. **i** MrGO-II. **j** MrGO-III. **k** MrGO-IV. **l** MrGO-V. **m** MXene. **n** MGO-AD. **o** rGO-AD. **p** MrGO-AD sheets. Scale bar, 5 μm (a-p, above) and 2 μm (a-p, below).

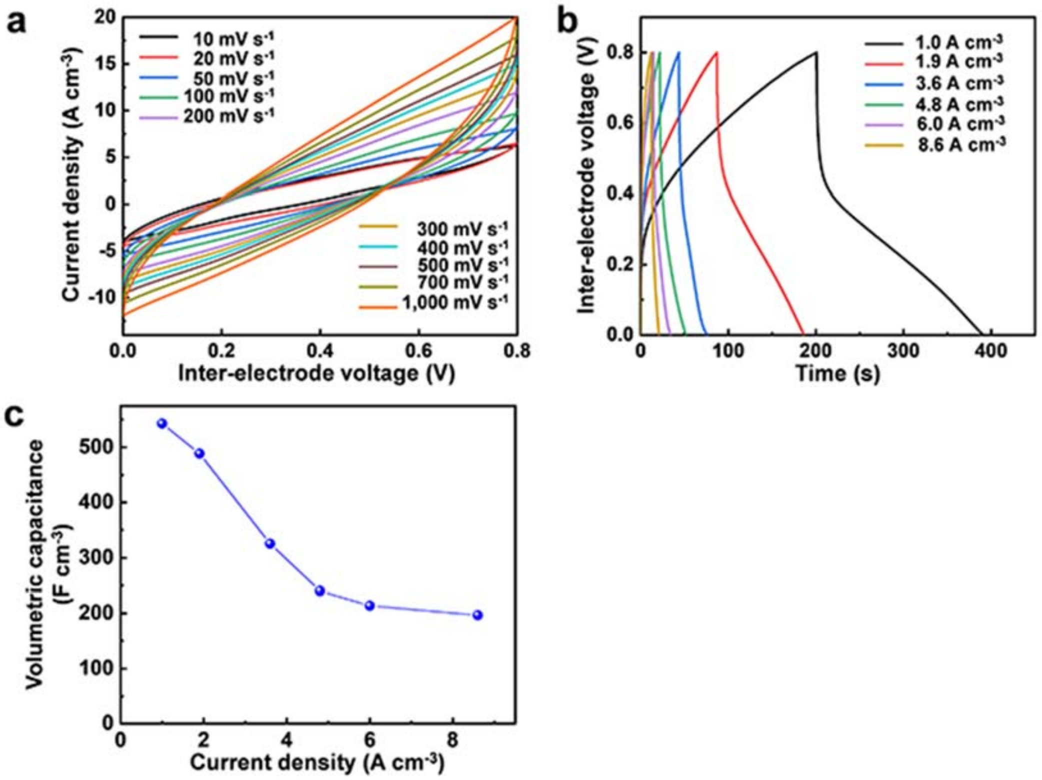

Supplementary Figure 30 | a CV curves at scan rates of 10 mV s^{-1} to 1,000 mV s^{-1} for a supercapacitor based on MrGO sheets. **b** Galvanostatic charge-discharge curves for a supercapacitor based on MrGO sheets. **c** Volumetric capacitance for current densities from 1.0 A cm^{-3} to 8.6 A cm^{-3} for a supercapacitor based on MrGO sheets.

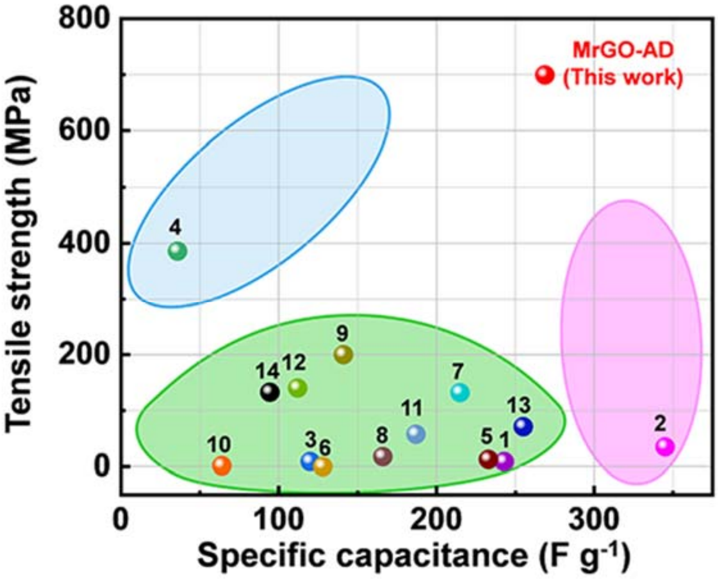

1 rGO/MnO_2 paper
2 rGO/polypyrrole paper
3 rGO/cellulose film
4 rGO wire/CNT fiber
5 rGO/PANI paper
6 rGO aerogel
7 rGO paper
8 Sulfonated aramid-graphene paper
9 Graphene/cellulose fiber
10 rGO/Mn_3O_4 film
11 rGO/aramid film
12 rGO/PANI fiber
13 rGO/CNT film
14 rGO/MXene fiber

Supplementary Figure 31 | Ashby plot of capacitance versus tensile strength, which compares a MrGO-AD sheet with other notable graphene-based composites that are in the literature.

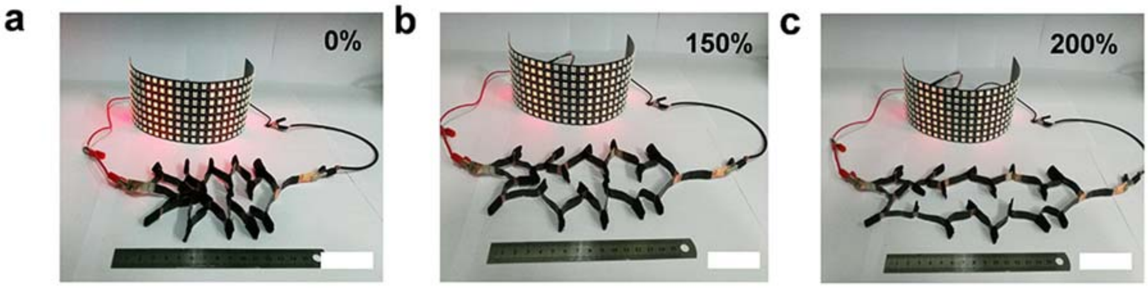

Supplementary Figure 32 | a–c An array of MrGO-AD sheet supercapacitors are connected to forms a network (including both in-parallel and in-series connections) that light a panel comprising an array of 256 LEDs with ~5V. This array of supercapacitors is cantilevered so that it can perform while stretched by up to 200%. Scale bar, 4 cm (a-c).

Supplementary Tables

Supplementary Table 1 | The weight percents of MXene and AD in GO-based sheets, which are derived from TGA curves.

Sample	MXene content (wt%) by TGA	AD content (wt%) by TGA
**MGO-I**	5.5	0
**MGO-II**	9.9	0
**MGO-III**	17.7	0
**MGO-IV**	29.9	0
**MGO-V**	45.1	0
**MrGO-AD**	19.0	2.0
**rGO-AD**	-	1.9

Supplementary Table 2 | The d-spacings of the neat GO, MGO, and MrGO sheets before and after reduction by HI.

Sample	d (Å)		d (Å)	
	Before reduction	2 θ (°)	After reduction	2 θ (°)
**GO**	7.53	11.74	3.69	24.09
**MGO-I**	7.77	11..38	3.68	24.17
**MGO-II**	7.82	11.31	3.65	24.38
**MGO-III**	7.97	11.09	3.64	24.45
**MGO-IV**	8.40	10.51	3.66	24.31
**MGO-V**	8.50	10.40	3.71	23.96
**MXene**	14.64	6.30	-	-
**MGO-AD**	7.99	11.05	3.68	24.14
**rGO-AD**	-	-	3.68	24.14

Supplementary Table 3 | The ratio of I_D to I_G for the investigated sheets, where I_D and I_G are the Raman intensities of the D and G peaks.

Sample	I_D:I_G
**GO**	0.9
**rGO**	1.6
**MGO**	1.2
**MrGO**	1.6
**MGO-AD**	1.2
**MrGO-AD**	1.6

Supplementary Table 4 | The percentage of C-Ti, C-C/C-H, C-O, and –COO/C-F according to the C 1s peak in the obtained XPS spectra.

Samples	C-Ti (%)	C-C/C-H (%)	C-O (%)	-COO/C-F (%)
**GO**	-	40.7	51.7	7.6
**MGO**	4.7	58.6	30.2	7.2
**MrGO**	3.8	80.3	9.9	5.9
**MrGO-AD**	3.7	81.6	9.5	5.3

Supplementary Table 5 | The full width at half maximum (FWHM) for the dependence of the intensity of WAXS on azimuthal angle. The thereby derived degree of orientation of graphene platelets is also provided for the rGO, rGO-AD, MrGO, and MrGO-AD sheets.

**Sample**	**FWHM (*θ*) (º)**	**Difference (º)**	**Degree of orientation (*δ*) (%)**
**rGO**	36.3	0	79.8
**MrGO**	27.1	9.2	84.9
**rGO-AD**	26.5	9.8	85.3
**MrGO-AD**	26.1	10.2	85.5

Supplementary Table 6 | The tensile strength and strain to failure for sheets of neat GO, MGO, and MrGO having different weight contents of MXene nanosheets.

Sample	MXene content (wt%) by TGA	Tensile strength (MPa)		Strain (%)	
		Before HI reduction	After HI reduction	Before HI reduction	After HI reduction
**GO**	-	82.2 ± 1.8	165.8 ± 1.1	2.6 ± 0.3	3.7 ± 0.3
**MXene**	100	61.2 ± 2.3	-	2.3 ± 0.5	-
**MGO-I**	5.5	144.3 ± 1.6	266.9 ± 6.9	2.1 ± 0.2	2.2 ± 0.2
**MGO-II**	9.9	179.7 ± 8.5	284.0 ± 11.8	3.1 ± 0.3	3.9 ± 0.3
**MGO-III**	17.7	226.3 ± 4.3	379.2 ± 2.5	4.4 ± 0.7	7.2 ± 0.3
**MGO-IV**	29.9	188.9 ± 2.1	302.0 ± 10.1	2.7 ± 0.3	3.0 ± 0.2
**MGO-V**	45.1	129.4 ± 3.7	170.2 ± 5..4	1.9 ± 0.2	2.4 ± 0.1
**MGO-AD**	-	433.5 ± 11.1	699.1 ± 30.6	7.8 ± 0.5	12.0 ± 0.7
**rGO-AD**	-	-	510.4 ± 12.4	-	7.1 ± 0.6
**MrGO-AD-m**	-	-	292.3 ± 4.9	-	5.4 ± 0.3

Supplementary Table 7 | The toughness for sheets of neat GO, MGO, and MrGO having different weight content of MXene.

Sample	Toughness (MJ m^{-3})	
	Before reduction	After HI reduction
**GO**	0.9 ± 0.1	2.4 ± 0.4
**MXene**	0.7 ± 0.01	-
**MGO-I**	1.2 ± 0.1	2.8 ± 0.4
**MGO-II**	3.2 ± 0.4	5.6 ± 0.6
**MGO-III**	6.2 ± 1.1	14.2 ± 1.7
**MGO-IV**	2.5 ± 0.3	4.5 ± 0.3
**MGO-V**	1.1 ± 0.1	2.2 ± 0.3
**MGO-AD**	15.3 ± 2.2	42.7 ± 3.4
**rGO-AD**	-	17.9 ± 0.9
**MrGO-AD-m**	-	7.3 ± 0.3

Supplementary Table 8 | The electrical conductivity of sheets of neat rGO, MGO, MrGO, rGO-AD, and MrGO-AD having different weight percents of MXene.

Sample	Electrical conductivity (S cm^{-1})
**MXene**	4,923.5 ± 15.0
**rGO**	335.8 ± 5.3
**MrGO-I**	412.7 ± 11.5
**MrGO-II**	509.8 ± 7.2
**MrGO-III**	1,036.6 ± 5.4
**MrGO-IV**	1,354.2 ± 41.1
**MrGO-V**	1,919.2 ±35.1
**MrGO-AD**	1,329.0 ± 15.9
**rGO-AD**	395.3 ± 0.6

Supplementary Table 9 | Comparison of the tensile strength, toughness, and electrical conductivity of MrGO-AD sheets with that for other graphene-based sheets.

Number	Samples	Tensile strength (MPa)	Toughness (MJ m^{-3})	Electrical conductivity (S cm^{-1})	Ref
1	rGO-PB	8.4	0.01	2	24
2	rGO-FPEG	45	none	0.034	25
3	GO-PVA	80.2	0.1	none	26
4	rGO-PVA	188.9	2.52	52.65	27
5	GO-PMMA	148.3	2.35	none	27
6	rGO-PAPB	382	7.5	337	28
7	GO-CNC	490	4	none	29
8	rGO-CNC	655	3.9	50	30
9	GO-Zn^{2+}	142.2	0.32	none	30
10	GO-Ca^{2+}	125.8	0.31	none	31
11	GO-Mg^{2+}	80.6	0.13	none	32
12	GO-Al^{3+}	100.5	0.23	none	32
13	GO-SL	300	2.2	none	33
14	rGO-SL	300	2.8	13.5	33
15	GO-AA-SCMC	305.0	8.2	none	34
16	GO-Al_2O_3-PVA	143.0	9.2	none	35
17	rGO-DWNT-PCDO	374.1	9.2	394.0	36
18	rGO-MMT-PVA	356.0	7.5	136.4	37

Number	Samples	Tensile strength (MPa)	Toughness (MJ m^{-3})	Electrical conductivity (S cm^{-1})	Ref
19	GO-MMT-SPVA	250.0	2.7	326.0	38
20	rGO-MoS_2-TPU	235.3	6.9	46.4	39
21	rGO-CS-Cu^{2+}	868.2	14.0	234.8	40
22	rGO-WS_2-PCDO	413.6	17.7	197.1	41
23	rGO–CNC–Cd^{2+}	419.4	11.0	152.4	42
24	rGO-PDA-Ni^{2+}	417.2	19.5	144.5	43
25	rGO-CMC-Mn^{2+}	475.2	6.6	297.1	44
26	rGO–Zn^{2+}–PCDO	439.1	7.5	120.8	45
27	rGO-AP/PSE-PCO	944.5	20.6	512.3	46
28	rGO-HPC-Cu^{2+}	274.3	6.7	127.7	47
29	rGO-NFC-PCDO	314.6	9.8	162.6	48
30	rGO-PAAP-Eu^{3+}	112.1	1.7	21.2	49
31	rGO-AD	458.2	9.8	430.0	17
32	πBG	1054	36	1192	20
**33**	**MrGO-AD**	**699.1**	**42.7**	**1329.0**	**This work**

Supplementary Table 10 | Comparison of the specific capacitance and tensile strength of the MrGO-AD sheets with that for other graphene-based sheets.

NO.	Samples	Preparation method	Capacitance F g^{-1} (F cm^{-3})	Tensile strength (MPa)	Ref
1	rGO/MnO_2 paper	Vacuum filtration	243	8.79	50
2	rGO/polypyrrole nanofiber paper	Vacuum filtration	345	35	51
3	rGO/cellulose	Vacuum filtration	120	8.67	52
4	rGO wire/CNT composite	Wet spinning	35.9	385.7	53
5	rGO/polyaniline	Electropolymerization	233	12.6	54
6	rGO aerogel	Supercritical CO_2 drying	128	0.15	55
7	rGO paper	Vacuum filtration	215	132	56
8	Sulfonated aramid-graphene paper	Vacuum filtration	166	17.5	57
9	Graphene/cellulose fiber	Wet spinning	141.1	199.8	58
10	rGO/Mn_3O_4 films	Electrochemical deposition	64	1.4	59
11	rGO/aramid	Vacuum filtration	187	57.5	60
12	rGO/PANI	Wet spinning	112 (148 F cm-3)	140	61
13	rGO/CNT	Continuous centrifugal casting	255 (407 F cm^{-3})	71	62
14	rGO/MXene	Wet spinning	94.4	132.5	63
15	rGO/melamine	Vacuum filtration	197.3 (F cm^{-3})	45	S9
**16**	**MrGO-AD**	**Vacuum filtration**	**268.8 (645.2 F cm^{-3})**	**~699.1**	**This work**

Supplementary Table 11 | Comparison of the energy density, power density, and tensile strength of MrGO-AD sheets supercapacitors with that for other materials used in supercapacitors.

Samples	Energy density ($mWh\ cm^{-3}$)	Power density ($mW\ cm^{-3}$)	Tensile strength (MPa)[a]	Calculation with Packaging	Ref
Graphene/cellulose fiber	~5.1	~500	199.8 (4.8 %)	NO	58
rGO/SWNT fibers	~6.3	~1200	84 (3.4%)	NO	64
rGO/PANI	~8.8	~350	140 (31%)	NO	61
rGO/CNT	~9.98	~400	71	NO	62
rGO/CNT composite	~3.4	~700	385.7	NO	53
rGO/aramid	~4.3	~600	57.5 (0.87%)	NO	60
rGO/MXene fiber	~5.1	~1693	132.5 (2.9%)	NO	63
5.5 V/100 mF commercial SC	~0.52	~350	-	YES	64
2.75 V/44 mF commercial AC-SC	~0.65	~900	-	YES	65
3 V/300 μF Al electrolytic capacitor	~0.0014	~45,000	-	YES	65
Li thin film battery	~10.0	~5.2	-	NO	65
**MrGO-AD**	**~13.0**	**~1,991.9**	**~699.1 (12.0%)**	**NO**	**This work**

a, The data in parentheses are the failure strain that corresponds to the reported tensile strength.

Supplementary Note 1

Component content calculated by TGA

M_{MXene}, M_{GO}, M_{rGO}, M_{AD}, M_{MGO}, $M_{MrGO\text{-}III,}$ and $M_{MrGO\text{-}AD}$ are the fractions of weight loss for MXene, GO, rGO, AD molecules, MGO, MrGO-III, and MrGO-AD sheets that are used in Supplementary equations (5) and (6). The weight content (W_1) of MXene in the MGO sheets was calculated using Supplementary equation (5). The results are in Supplementary Table 1.

$$w_{1(MXene/MGO)}=\frac{M_{MGO}\text{-}M_{GO}}{M_{MXene}\text{-}M_{GO}} \tag{5}$$

$$w_{2(MrGO\text{-}AD\ -\ MrGO\text{-}III)}=\frac{M_{MrGO\text{-}AD}\text{-}M_{MrGO}}{M_{AD}\text{-}M_{MrGO}} \tag{6}$$

Supplementary Note 2

Calculation of the orientation

The degree of graphene platelet orientation (δ) was calculated using the following Supplementary equation (7).

$$\delta = \frac{180^\circ - FWHM}{180^\circ} \times 100\% , \tag{7}$$

where FWHM is the full width at half maximum of X-ray scattering intensity as a function of the azimuthal angle.

Supplementary Note 3

Calculation of the porosity

We used the following Supplementary equation (8) to calculate the porosity (w):

$$w=\frac{V_0-V}{V_0}=\frac{V_0-V_G-V_{AD}-V_M}{V_0} \tag{8}$$

where V_0 is the sample volume, V is the total molecular volume of the materials in the sample, and V_G, V_{AD}, V_M correspond to the molecular volumes of graphene, AD molecules, and MXene (which are derived from the densities of the graphene, AD, and MXene).

Density and porosity of sheets.

**Sample**	**Density (g cm^{-3})**	**Porosity (%)**
rGO	1.95	15.2
rGO-AD	2.03	8.0
MrGO	2.30	5.2
MrGO-AD	2.35	4.0

Supplementary Note 4

Procedure of the stretching by MD simulations

According to MD simulations **(Supplementary Fig. 26b)**, when the stretching procedure starts, the microcrack of adjacent rGO platelets, attributed to the breakage of weak interactions along with mutual sliding of the rGO platelets, is initiated **(i to ii)**. With increasing loading to stretch, MXene nanosheets slide across each other due to the strong Ti-O-C bonding covalently crosslinked with rGO platelets. Meanwhile, the long-chain AD molecules crosslinking with rGO plateletsvia π-π bridging interaction are stretched and further arrest crack propagation before completely fracturing **(ii to iv)**. As a result, the MXene nanosheets are separated from each other and the AD molecules are broken. With further stretching, the Ti-O-C covalent bonding between MXene nanosheets and rGO platelets is broken to arrest crack propagation for promoting toughness until complete fracture of the sheets **(Step iv to v)**. In addition, the tensile strength of simulations **(Supplementary Fig. 26a)** is higher than the experimental value, which is due to the idealized model of simulations.

Supplementary References

1. Plimpton, S. Fast parallel algorithms for short-range molecular dynamics. *J. Comput. Phys.* **117**, 1–19 (1995).
2. Hockney, R. W. & Eastwood, J. W. *Computer simulation using particles*. (Taylor & Francis, Inc., 1981).
3. Berendsen, H. J. C., Postma, J. P. M., van Gunsteren, W. F., DiNola, A. & Haak, J. R. Molecular dynamics with coupling to an external bath. *J. Chem. Phys.* **81**, 3684–3690 (1984).
4. Vanderbilt, D. Soft self-consistent pseudopotentials in a generalized eigenvalue formalism. *Phys. Rev. B* **41**, 7892–7895 (1990).
5. Perdew, J. P. & Zunger, A. Self-interaction correction to density-functional approximations for many-electron systems. *Phys. Rev. B* **23**, 5048–5079 (1981).
6. Perdew, J. P., Burke, K. & Ernzerhof, M. Generalized gradient approximation made simple. *Phys. Rev. Lett.* **77**, 3865–3868 (1996).
7. Dion, M., Rydberg, H., Schröder, E., Langreth, D. C. & Lundqvist, B. I. Van der waals density functional for general geometries. *Phys. Rev. Lett.* **92**, 246401–246404 (2004).
8. Kresse, G. & Furthmüller, J. Efficient iterative schemes for *ab initio* total-energy calculations using a plane-wave basis set. *Phys. Rev. B* **54**, 11169–11186 (1996).
9. Wang, G. et al. Graphene Thin films by noncovalent-Interaction-driven assembly of graphene monolayers for flexible supercapacitors. *Chem* **4**, 1–15 (2018).
